# Disruptive viability selection on a black plumage trait associated with dominance

**DOI:** 10.1111/jeb.12717

**Published:** 2015-09-14

**Authors:** P. Acker, A. Grégoire, M. Rat, C. N. Spottiswoode, R. E. van Dijk, M. Paquet, J. C. Kaden, R. Pradel, B. J. Hatchwell, R. Covas, C. Doutrelant

**Affiliations:** ^1^ CEFE UMR 5175CNRS – Université de Montpellier – Université Paul‐Valéry Montpellier – EPHEMontpellier Cedex 05France; ^2^ Laboratoire Évolution & Diversité Biologique (UMR 5174 EDB), Université Toulouse 3 Paul Sabatier – CNRS ‐ ENFAToulouseFrance; ^3^ Percy FitzPatrick InstituteDST‐NRF Centre of ExcellenceUniversity of Cape TownRondeboschSouth Africa; ^4^ Department of ZoologyUniversity of CambridgeCambridgeUK; ^5^ Department of Animal and Plant SciencesUniversity of SheffieldSheffieldUK; ^6^ The Royal Zoological Society of ScotlandEdinburgh ZooEdinburghUK; ^7^ CIBIOUniversity of PortoVairãoPortugal; ^8^ Biology DepartmentScience FacultyUniversity of PortoPortoPortugal

**Keywords:** badge of status, capture–recapture, fitness, fluctuating selection, individual variation, longitudinal study, melanin, mixed models, social selection

## Abstract

Traits used in communication, such as colour signals, are expected to have positive consequences for reproductive success, but their associations with survival are little understood. Previous studies have mainly investigated linear relationships between signals and survival, but both hump‐shaped and U‐shaped relationships can also be predicted, depending on the main costs involved in trait expression. Furthermore, few studies have taken the plasticity of signals into account in viability selection analyses. The relationship between signal expression and survival is of particular interest in melanin‐based traits, because their main costs are still debated. Here, we first determined the main factors explaining variability in a melanin‐based trait linked to dominance: the bib size of a colonial bird, the sociable weaver *Philetairus socius*. We then used these analyses to obtain a measure representative of the individual mean expression of bib size. Finally, we used capture–recapture models to study how survival varied in relation to bib size. Variation in bib size was strongly affected by year and moderately affected by age, body condition and colony size. In addition, individuals bearing small and large bibs had higher survival than those with intermediate bibs, and this U‐shaped relationship between survival and bib size appeared to be more pronounced in some years than others. These results constitute a rare example of disruptive viability selection, and point towards the potential importance of social costs incurred by the dominance signalling function of badges of status.

## Introduction

Long‐term studies give insight into fluctuations in the strength, direction and shape of the associations between traits and fitness in nature. They are essential to assess the biological importance of the conclusions obtained with short‐term experiments and are extremely valuable for determining the complexity underlying trait variability and plasticity (Svensson & Gosden, 2007; Cornwallis & Uller, 2010). Animal signals have these features of complex, plastic traits. They are intrinsically positively linked to fitness owing to their role in intraspecific competition and cooperation, sexual and nonsexual social mate choice, and individual, sexual or species recognition (Andersson, 1994; Maynard Smith & Harper, 2003; Searcy & Nowicki, 2005; Hill & McGraw, 2006).

Short‐term experiments and analyses of long‐term data have demonstrated associations between animal signal expression and reproductive success in a broad range of taxa. By contrast, the association between signal expression and survival is still far from being well understood, particularly in the long term (Grégoire *et al*., 2004; Figuerola & Senar, 2007; Meunier *et al*., 2011; McCullough & Emlen, 2013). Previous studies have mainly investigated linear relationships between signals and survival, but more complex quadratic and temporally variable relationships are predicted, depending on the signals' main functions and costs of production and maintenance, as well as on the environmental and social conditions experienced.

A negative quadratic correlation (i.e. hump‐shaped relationship) between survival and signalling can be predicted for condition‐dependent signals (indicative of stabilizing viability selection; Grégoire *et al*., 2004; Figuerola & Senar, 2007). Under the hypothesis of condition dependence, signalling and/or cheating have intrinsic production or maintenance costs for the emitter of the signal (Zahavi, 1975; Grafen, 1990; Searcy & Nowicki, 2005) and low‐quality individuals, which are expected to have lower chances of survival, should produce poorly developed signals, whereas better‐quality individuals should produce more developed signals and have higher chances of survival. As a result, a positive correlation between survival probability and signal size is expected (see Jennions *et al*., 2001 for a meta‐analysis of studies mostly testing linear relationships between these traits). However, at some point, this correlation should reverse because individuals with more developed signals might be also more detectable and/or have a lower ability to escape predators and thus might suffer higher mortality due to predation (e.g. Stuart‐Fox *et al*., 2003; Basolo & Wagner, 2004). In addition, as a result of trade‐offs between investment in costly signal production and self‐maintenance, individuals bearing the more developed signals might die earlier (e.g. Hunt *et al*., 2004; Preston *et al*., 2011). Taken together, these processes should result in stabilizing viability selection for condition‐dependent signals.

For signals that are predicted by theory to have social costs (i.e. costs imposed by their receivers, not by the production of the signal; Maynard Smith & Harper, 1988, 2003; Searcy & Nowicki, 2005), the expected relationship with survival is likely to differ from that predicted for the condition‐dependent signals mentioned above. Specifically, disruptive selection may be expected. For example, for badges of status (i.e. traits that signal social status in a group), which are commonly found in many taxa (e.g. in insects, fish, lizards, birds or mammals; Whiting *et al*., 2003; Tibbetts & Dale, 2004; Senar, 2006; Bergman *et al*., 2009; Johnson & Fuller, 2015; Bro‐Jørgensen & Beeston, 2015), social costs should arise because individuals with similar badges are expected to interact aggressively, whereas individuals presenting dissimilar badges are predicted to accept a hierarchy based on badge size (Rohwer, 1977). Because badge sizes are typically normally distributed in a population, and because disputes are more difficult to settle passively among individuals with the same badge size (Maynard Smith & Harper, 1988, 2003; Senar, 2006), the more numerous individuals with intermediate badge sizes are predicted to have a higher probability of engaging in aggressive interactions. Consequently, individuals with intermediate badge sizes may suffer higher costs of aggressive interactions and have lower survival. Thus, badge sizes as signals of social status could be under disruptive viability selection, that is have a positive quadratic (U‐shaped) relationship with survival. Yet, to our knowledge, this prediction has never been tested.

Social behaviour, physiology and condition are, however, often linked and appear to have complex and dynamic two‐way interactions (e.g. Safran *et al*., 2008). As a result of these interactions, signals may bear physiological costs in addition to social costs, and these physiological costs may in some cases be condition dependent (see Tibbetts, 2014 for a review of these ‘integrative costs’). For instance, in the pūkeko, *Porphyrio porphyrio melanotus*, an experimental decrease in apparent red shield size caused both an increase in the amount of aggression received (i.e. a social cost) and a decrease in true shield size due to a hormonal change arising from the higher level of aggression received (Dey *et al*., 2014). Furthermore, some signals can have several functions. For instance, badges of status may not only serve to establish dominance, but also be used in subsequent mate choice (Berglund *et al*., 1996; Qvarnström & Forsgren, 1998). Such signals may experience both social costs and intrinsic production or maintenance costs dependent on the condition of the emitter, in which case the prediction of a U‐shaped correlation between badge size and survival should only be realized when social costs overcome the other costs associated with condition dependence. Additionally, this association between signals and survival is likely to fluctuate according to the prevailing social and climatic conditions, and hence to vary through time.

The costs of signals used in competitive interactions remain poorly understood (McCullough & Emlen, 2013). Black badges are especially interesting to test the predictions above and improve our understanding of the associations between agonistic signals and survival. Black coloration (i.e. melanin‐based pigment; Fox, 1976; McGraw, 2006) has repeatedly been found to function primarily as a badge of status, and hence in competitive interactions in a wide range of taxa (e.g. in insects: Tibbetts & Dale, 2004; in lizards: Osborne, 2005; in birds: Senar, 2006; Tibbetts & Safran, 2009; in fish: Johnson & Fuller, 2015; in mammals: Bro‐Jørgensen & Beeston, 2015). In addition, although black coloration has long been considered an example of a colour signal with relatively low production costs and high social costs, more and more studies suggest that this colour signal could in fact be costly to produce, or linked to condition through pleiotropy (Senar, 2006; Griffith *et al*., 2006; Stoehr, 2006; McGraw, 2008; Ducrest *et al*., 2008; Roff & Fairbairn, 2013; Roulin, 2015).

Studying the link between survival and signalling might help to clarify the key factors ensuring the honesty of melanic badges. In a meta‐analysis of 15 bird studies, Meunier *et al*. (2011) found that the sign and magnitude of the relationship between survival and melanin‐based coloration is species or trait specific. However, this meta‐analysis included both polymorphic and monomorphic colour traits, and these are likely to have different functions and costs. Moreover, none of the studies included in this meta‐analysis tested for the quadratic relationships predicted above, and so more studies on badges of status are needed before general conclusions are made. In fact, quadratic relationships have very rarely been tested for in any type of signal (Jennions *et al*., 2001).

Another problem with previous studies is that most have used return rates as a proxy of survival. This is potentially problematic because the analysis of the relationship between survival and colour traits in natural populations requires capture–recapture (CR) tools to model survival and recapture probability, in order to avoid biases in survival estimates (Gimenez *et al*., 2008). Among the few CR studies investigating the link between a colour trait and long‐term survival (e.g. Jones *et al*., 2004; Bize *et al*., 2006; Roulin & Altwegg, 2007; Potti *et al*., 2013; Emaresi *et al*., 2014), only two tested for a quadratic relationship (Grégoire *et al*., 2004; Figuerola & Senar, 2007), and these involved carotenoid‐based signals which are assumed to be condition dependent and more closely linked to intersexual than intrasexual social interactions.

Plasticity is a major complication when studying relationships between signals and survival. Many signals change throughout an individual's life, either at discrete intervals (e.g. moult in birds: Hill & McGraw, 2006; fall of cervid antlers: Goss, 2012) or in a much more rapid and flexible way within short time periods (e.g. bare skin parts in birds: Hill & McGraw, 2006; amphibians: Nilsson Sköld *et al*. 2013; cephalopods: Mäthger *et al*., 2009; fishes: Kodric‐Brown, 1998; Nilsson Sköld *et al*. 2013). Such plasticity needs to be taken into account. Yet, to date, survival analyses avoid this issue by relating the coloration expressed in the first year of life to subsequent long‐term yearly survival (over the capture–recapture history), or by relating signal expression in a given year to short‐term yearly survival (i.e. survival from that year to the next).

In this study, we investigated the relationship between survival and a colour patch that has characteristics of a badge of status (Rat *et al*., 2015), the size of the black bib of a colonial passerine bird, the sociable weaver, *Philetairus socius*. The study is based on bib measures taken over 6 years, and capture–recapture data over 9 years. In sociable weavers, bib size is positively associated with social dominance and it changes when the rank of an individual changes (Rat *et al*., 2015). Furthermore, as expected for badges of status, medium‐ranked birds engaged more in aggressive interactions than high‐ranked individuals, suggesting that competition over resources is more pronounced among birds of intermediate social status (Rat *et al*., 2015). However, no information is currently available about the possible condition dependence of the black bib in this species and its role, if any, in mate choice.

We first examined the variability of bib size, using both population‐level and individual‐level (within‐ and between‐individual partitioning) analyses to estimate the effect of several factors known to influence signal expression in many other species: year, age, sex, body condition, colony identity and colony size. We then used this multivariate analysis with repeated measurements over time to obtain a measure reflecting the mean individual expression of bib size with the best linear unbiased predictors (BLUPs) of the individual random effects. This method allows the mean individual expression of a plastic trait to be estimated over the capture–recapture history (Bergeron *et al*., 2013). Finally, we investigated the relationship between bib size and survival, estimating both short‐term yearly survival (from 1 year to the next) in relation to the bib size expressed just before the survival event, and long‐term yearly survival (over the capture–recapture history) in relation to the mean expression of bib size.

Because of the potential variation in the relative magnitude of nonsexual and sexually selected social benefits of a large bib, and the possible social and intrinsic costs of producing and bearing that signal, we tested all possible relationships between survival and bib size, including directional, stabilizing and disruptive viability selection. In addition, as sociable weavers live in a semi‐arid region of the world where annual rainfall and temperature fluctuate greatly (Maclean, 1973; Covas *et al*., 2008), and climatic fluctuations are known to impact food availability, competition and investment in signals (Cockburn *et al*., 2008; Vergara *et al*., 2012), we tested for the potential of annual variation in the relationship between survival and bib size.

## Materials and methods

### Study species and study site

The sociable weaver is a colonial, cooperatively breeding passerine endemic to the semi‐arid savannahs of southern Africa (Maclean, 1973). Adults display a black bib which, according to Maclean (1973), is replaced within a month during the annual antero‐posterior body moult that follows the breeding season. There is no apparent sexual dimorphism: sexes are indistinguishable in the field and previous studies did not found significant sex differences in bib size and other plumage traits (Rat *et al*., 2015). The study site is at Benfontein Nature Reserve (28°52′S, 24°51′E), South Africa. The area is semi‐arid, experiencing low and unpredictable rainfall (average 431 ± 127 mm per year; South African Weather Service, Pretoria). The study area contains about 30 active colonies each year.

Birds were photographed when captured at the colonies in 2002–2004 and 2010–2012. Individuals were held lying on their back in a standardized position, alongside a ruler. We obtained absolute measures of bib size (cm²) by counting black pixels of the bib with Adobe Photoshop CS6 (Fig. S1). Most of the time (61% of the cases), several photographs (mean = 2.9 ± 0.53) of the same individual were taken at the same capture occasion, repositioning the feathers between photographs. Bib size was then estimated as the average value of the measures obtained from each of these photographs (see Appendix S1 for more details).

We included 888 measures of bib size from 662 individuals (176 individuals sampled twice and 25 sampled three times at different time points) in the analyses. Bib size was normally distributed with a mean of 1.40 ± 0.22 cm² (Fig. S2). Repeatability between measurements from photographs of the same bird taken on the same occasion (based on intraclass correlation coefficient; Nakagawa & Schielzeth, 2010) was high (*r *= 0.94, *F*_1028,1203_ = 33.01, *P *<* *0.001). All bib photographs were taken by CNS, RvD and MR, and measured by PA, MR and JCK (Tables S1 and S2). Measurements were highly repeatable between observers (30 photographs measured by two observers: repeatability *r = *0.97, *F*_29,30_ = 63.97, *P *<* *0.001).

Captures at the colonies were conducted in the field since July 1993 (Covas *et al*., 2011; Altwegg *et al*., 2014). Birds were captured by flushing them into mist nets erected around colonies at dawn. The few birds that escaped the capture were counted, enabling accurate estimations of colony size.

At capture, body mass and tarsus length were systematically measured and a blood sample was taken from the brachial vein. Sex was genetically determined for all individuals using standard molecular techniques (Griffiths *et al*., 1998). The exact age was known for 23% of the birds, which were those ringed as nestlings (until *ca*. 20 days after hatching) or as juveniles, that is before their adult plumage was complete (which occurs *ca*. 4 months after fledging). Individuals ringed for the first time as adults were also included in this study by assigning them a minimum possible age (as commonly applied, *e.g*. Hill, 1993; Brommer *et al*., 2007; Evans & Sheldon, 2013) of 4 months (time necessary to complete a bib after fledging) plus 20 days (nestling period) at first encounter. Measurements of incomplete bibs of nestlings and juveniles were not included in this study to avoid a pattern of variation with age due to early‐life plumage maturation.

### Variability in bib size

We studied variability in bib size using linear mixed models (LMMs) and model parameters were estimated by frequentist methods in R 2.15.2 (R Core Team, 2012).

#### Population‐level pattern of variation

A first set of models was developed to explore the population‐level pattern of variation in bib size in relation to age (from 5 to 143 months, but individuals over 120 months were grouped together, because there were only eight individuals and no obvious directional variation within this category), body mass, tarsus length, colony size (from 4 to 76 adults), sex and interactions between sex and each of the other variables. Year, colony and individual identity were additionally fitted as random effects. Body mass and tarsus length were always included together, to estimate body condition (Garcia‐Berthou, 2001).

Model selection followed a backwards stepwise procedure. First, the random effect terms were tested with likelihood‐ratio tests (LRTs). Then, the significance of fixed effects was evaluated using Markov chain Monte Carlo (MCMC) samples from the posterior distribution of the parameters (i.e. a Bayesian approach, assuming uninformative priors; Bolker *et al*., 2009) with 10^6^ simulations. Nonsignificant effects having *P*‐values > 0.1 were removed following MCMC‐based probabilities (*P*_MCMC_). To ensure the relevance of the selection process, all models were compared using the corrected Akaike information criterion (AICc, see Johnson & Omland, 2004). This comparison also included all possible models differing from the minimum model by the removal of one of the selected fixed effects. Marginal and conditional *R*² were computed (Nakagawa & Schielzeth, 2013) to yield estimates of the amount of variance explained. Lastly, LMM‐based standard and adjusted repeatabilities were calculated (see Nakagawa & Schielzeth, 2010) to improve our representation of the within‐ and between‐individual variation in bib size.

Graphical observation of the relationship between age and bib size suggested that a nonlinear relationship might offer a better fit to the data. We thus tested different ways of modelling the relation between age and bib size using the minimum model obtained before as a reference: we tested (i) a quadratic relationship, (ii) a replacement of age (linear) by its logarithm and (iii) a piecewise linear effect of age (with one breakpoint maximizing the likelihood; *e.g*. Toms & Lesperance, 2003).

We thereafter compared these three models to the minimum model selected previously by the backwards stepwise procedure, and retained the model with the lowest AICc. This model was then used to compute the BLUPs used in the survival analyses (see the corresponding section hereafter).

#### Within‐ and between‐individual pattern of variation

In standard mixed models, the estimates of fixed effects of continuous predictor variables reflect a combination of the within‐ and between‐individual effects which can neither be interpreted as the within‐ nor as the between‐individual effect, except when they are identical or when one of the two is null. Here, we used the within‐subject centring approach (van de Pol & Wright, 2009) to disentangle the between‐ and within‐individual effect of all continuous predictor variables potentially subject to within‐individual variation: age and age², colony size, mass. Further details and equations are given in Appendix S2.

### Survival in relation to bib size

We used capture–recapture (CR) models to estimate survival of marked individuals with the software e‐surge v1.8.5 (Choquet *et al*., 2009a), following a maximum‐likelihood procedure. CR models distinguish between the probability of local survival (*ϕ*) and the probability of recapture (*p*), and allow assessment of the effect of discrete and continuous covariates on these parameters (Lebreton *et al*., 1992). The overall goodness‐of‐fit test performed with u‐care v2.3.2 (Choquet *et al*., 2009b) indicated that the data met the Cormack–Jolly–Seber (CJS) assumptions (i.e. no trap dependence and no transient effect; *χ* ^2^ =36.62, *P *=* *0.22). Model selection relied on AICc (see Johnson & Omland, 2004).

Our aim was to explore the relationship between bib size and survival. The bib is renewed annually, such that bib size varies between years. When using CR models, we face a technical problem because we cannot infer the value of a plastic trait for missing data points. In addition, the mean individual expression of the trait (observed in the long‐term among all occasions) and punctual expression of the trait (observed in the short‐term on one occasion) might show different associations with survival. To tackle these problems, we used two measures of bib size, representing either the trait expressed in a given year, or the mean individual expression in the trait across all years for which measurements were obtained.

Bib size expressed in a given year was used to investigate the relationship between bib size and short‐term yearly survival (i.e. between two capture occasions). It corresponds to photographs taken in the field the year before survival estimation (only the first photograph was used for individuals that were caught and photographed during several years). We used a standardized measure of bib size (SB), further standardized within each year, because birds are likely to use the trait value relative to the other birds in the population in each year, rather than using the absolute value of the trait. This expectation was confirmed by similar analyses with the untransformed measure of bib size, which showed that the models did not fit the data as well (results shown in Appendix S4, Table S4). We only related SB to short‐term yearly survival, because of the within‐individual variability in the trait.

The mean individual expression of bib size was employed to investigate the relationship between bib size and long‐term yearly survival (i.e. over the entire capture–recapture history). It corresponds to mean‐adjusted bib sizes (MAB), which are the individual random effects obtained with the final model retained to describe the variability in bib size (see population‐level analyses). These values are the BLUPs of the individual random effects (i.e. the individual conditional means). They represent an individual's mean deviation from the overall intercept given the data and the significant covariates included in the model (Pinheiro & Bates, 2000). Importantly, this mean individual investment is free of the known significant sources of environmental variation that were included in the model retained to describe the variability in bib size (year, age, mass, colony size, colony identity). This method was also applied by Bergeron *et al*. (2013) to achieve a similar goal. BLUPs constitute a useful tool to investigate the relationship with survival in the long term, because they handle missing values in longitudinal measurements and can be applied to all the capture–recapture history of any individual.

We only considered CR models with an *a priori* biological interpretation (Burnham & Anderson, 1998). First, we generated models in which survival and recapture probabilities were either time dependent or constant. Then, the effect of sex was added. Finally, bib size was added with either an effect of standardized bib size (SB) on short‐term yearly survival, or an effect of MAB on long‐term yearly survival. Bib size was included as a linear and/or a quadratic component. Quadratic effects of bib size were considered alone (i.e. without the linear effect) when the linear effect of bib size was not significant and not meaningful (i.e. when its inclusion did not affect the relationship between bib size and survival). The removal of the linear term imposes symmetry to the relationship, centred on the mean of bib size. We always tested for an interaction between time and bib size, sex and bib size, and a three‐way interaction between time, sex and bib size.

## Results

### Variability in bib size

#### Population‐level pattern of variation

**Figure 1:**
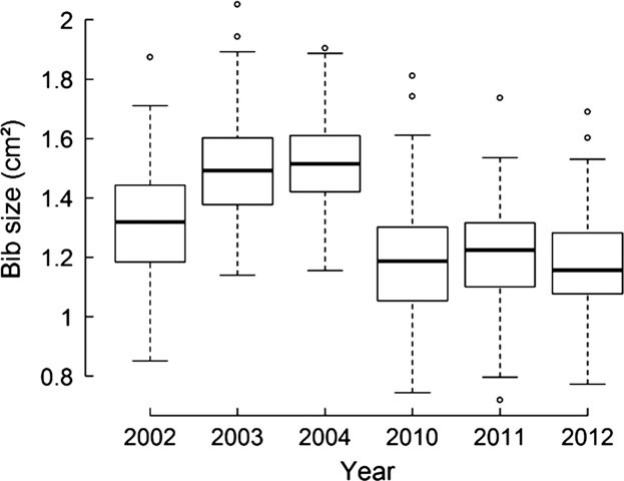
Variation in bib size for the 6 years of measure. Bib size was adjusted by the other effects of the predictor variables included in the linear mixed model.

**Figure 2:**
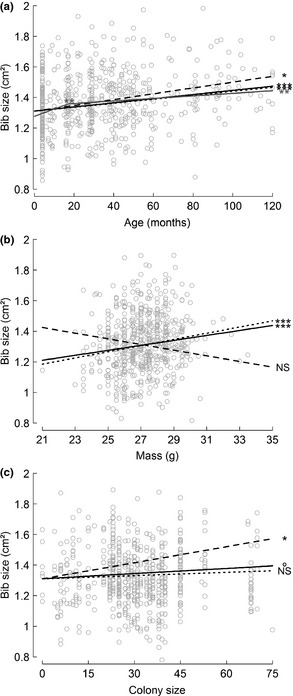
Population level, between‐ and within‐individual effect on bib size of (a) age, (b) mass and (c) colony size. In each plot, bib size was adjusted by the effects of the other predictor variables included in the minimum model. The within‐individual effect is represented by a black dashed line, the between‐individual effect by a black dotted line and their combined effect at the population‐level by a black bold line. In plot (a), the additional grey segments give the combined within‐ and between‐individual effect of age at the population level from the final model with a piecewise relation. Black stars stand for significance level according to *P*_MCMC_ (° = 0.1, *: 0.05, **: 0.01, ***: 0.001, NS: nonsignificant)

The random effects of year, colony and individual identity on bib size were highly significant (LRT: *P* < 0.001 for year and colony, *P* = 0.004 for individual; Fig. 1). Bib size was positively associated with body mass and age (both *P*_MCMC_ < 0.001; Fig. 2a,b, Table 1). Males had slightly larger bibs than females, but the difference was minimal (+0.032 cm², *P*_MCMC_ = 0.015, Table 1). Bib size tended to be positively correlated with colony size (*P*_MCMC_ = 0.089; Fig. 2, Table 1), and all other effects included in the model were not significant (*P*_MCMC_ > 0.21, Appendix S3). AICc values (Table S3) did not contradict the selection of the meaningful effects with *P*_MCMC_.

**Table 1:** Decomposition of the significant fixed effects into their respective within‐ and between‐individual effects in the minimum model selected to describe variability in bib size. Estimates *β*_*c*_ from the standard mixed model equation are a combination of the within‐ and between‐individual effects. Applying the within‐individual centring approach, estimates *β*_*w*_ are the within‐individual effects and *β*_*b*_ are the between‐individual effects. A variant of this latter approach allows tests for a significant difference between both effects (*β*_*b*_ − *β*_*w*_). *P*_MCMC_ are *P*‐values based on the posterior distribution from MCMC samples. See Materials & Methods and Appendix S2 for details

Effect	Parameter	Estimate ± SE	*P*_MCMC_
Sex	*β*_*c*_	0.032 ± 0.013	0.015
Age	*β*_*c*_	0.001 ± 0.000	< 0.001
	*β*_*w*_	0.002 ± 0.001	0.017
	*β*_*b*_	0.001 ± 0.000	< 0.001
	*β*_*b*_ *–β*_*w*_	−0.001 ± 0.001	0.46
Mass	*β*_*c*_	0.016 ± 0.005	< 0.001
	*β*_*w*_	−0.019 ± 0.014	0.23
	*β*_*b*_	0.020 ± 0.005	< 0.001
	*β*_*b*_ *–β*_*w*_	0.039 ± 0.015	0.014
Colony size	*β*_*c*_	0.001 ± 0.001	0.089
	*β*_*w*_	0.004 ± 0.001	0.013
	*β*_*b*_	0.001 ± 0.001	0.29
	*β*_*b*_ *–β*_*w*_	−0.003 ± 0.001	0.051

Most of the variance in bib size was explained by the random effects (year, colony and individual identity) and the fixed effects (age, body mass and tarsus length and colony size) together (conditional R²: $R_{c}^{2}$ = 0.542), yet fixed effects alone explained a very small part of the variance (marginal R²: $R_{m}^{2}$ = 0.063). The values of R², AICc and the estimated variance of the year effect suggested that year was the most important of the explanatory variables explaining the variation in bib size (Fig. 1, Table S3). Within‐individual repeatability of bib size was significant but low: *r *=* *0.37 ± 0.06 (*F*_661,226_ = 1.80, *P *<* *0.001) for standard anova‐based repeatability, and *r *=* *0.19 (*P* = 0.002 from LRT) for LMM‐based adjusted repeatability in the final model that accounted for effects of year, age, condition, colony size, colony identity and individual identity.

Models including a nonlinear relationship with age (logarithm and piecewise regressions) provided better AICc values than the minimum model described above (ΔAICc > 2). The piecewise model including a nonlinear relationship with age was considered to be the most reliable to extract the BLUPs of the individual random effects (to compute MABs). This model had a piecewise regression with a breakpoint at the age of 17 months (Fig. 2), showing that the age effect was positive and significant both before and after the breakpoint (*P*_MCMC_ < 0.001 and *P*_MCMC_ = 0.006, respectively), yet the slope before 17 months was six times higher than the slope after 17 months (Fig. 2). The effects of other variables were similar to those found in the minimum model previously described.

#### Within‐ and between‐individual pattern of variation

The between‐ and within‐individual effects of age on bib size were significantly positive and did not differ significantly, meaning that bib size increased with age during a bird's life (Fig. 2, Table 1). Moreover, age did not have any between‐ or within‐individual quadratic effect (*P*_MCMC_ > 0.36), indicating a continuous increase of bib size with age.

Bib size varied significantly with mass (positive effect) between individuals but not within individuals, and these between‐ and within‐individual effects were significantly different (Fig. 2, Table 1). This means that bib size did not change with the body condition of an individual, but that among different individuals, those with better body condition had larger bibs.

The within‐individual effect of colony size on bib size was significantly positive (*P*_MCMC_ = 0.013), unlike the corresponding between‐individual effect (*P*_MCMC_ = 0.29), and these effects were not significantly different from one another (*P*_MCMC_ = 0.051; Fig. 2, Table 1). Thus, birds that experienced a change in colony size also changed their bib size, yet independently of its size, each colony contained individuals with both large and small bibs.

### Survival in relation to bib size

The ‘null model’ included time dependence for both recapture probabilities and survival probabilities, with both probabilities varying substantially among years (between 0.52 and 0.84 for recapture and 0.56 and 0.76 for survival). The model also included an additive effect of sex on survival, with males having higher survival than females.

**Figure 3:**
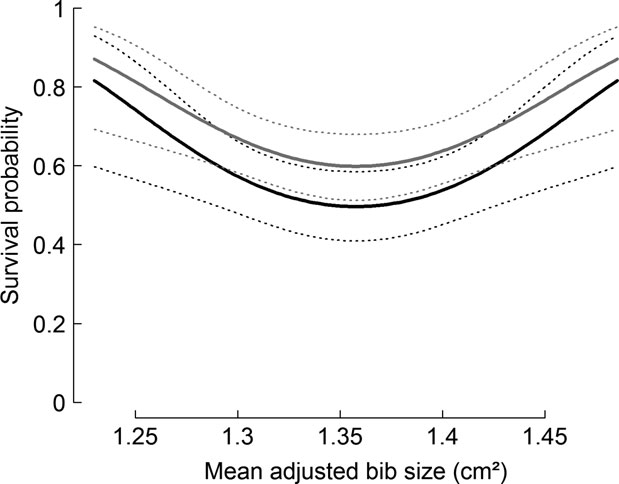
Yearly survival probability according to mean‐adjusted bib size. The plotted lines represent estimated survival probabilities obtained with the best model without interaction between time and bib size (*ϕ*_t+_ _MAB__²+sex_,*p*_t_), which indicates a significant pattern of disruptive viability selection over the data set. Females are plotted in black and males in grey. The solid lines indicate the means and dotted lines 95% confidence interval. In this model, there was an additive effect of time on survival. Here, we plotted the relationship for 2003–2004, but this convex relationship is more pronounced in years with lower survival, and less pronounced in years with higher survival (see Fig. S3 for the other time steps).

We obtained six models which performed better than this null model (∆AICc ≥ 2, Table 2; see Table S4 for all models). All of these models included a significant positive quadratic effect of bib size on survival, that is showed a U‐shaped relationship (Fig. 3). Five of these selected models showed an effect of MAB on long‐term yearly survival, and one showed an effect of SB on short‐term yearly survival. These models indicated that birds with small and large bibs had higher survival than birds with intermediate bib sizes (i.e. are indicative of disruptive viability selection).

**Table 2:** First ten best models for the viability selection of bib size. MAB (mean‐adjusted bib size obtained from BLUPs) was related to long‐term yearly survival, and SB (standardized bib size produced one year) was related to short‐term yearly survival (survival the year after). The notation used is the general notation of Lebreton *et al*. (1992): *ϕ* stands for survival and *p* for recapture probability. *K* corresponds to the number of parameters. ΔAICc is used to compare any model with the best model, whereas Δ_0_AICc is used to compare any model with the ‘null model’ (i.e. the best model without any effect of bib size on survival, *ϕ*_t+sex_,*p*_t_). The rank gives the descending order of AICc among the models. AICcW is the AICc weights. 1st(…) = effect present only during first year of capture–recapture history (i.e. after the first photograph was taken)

Variables included	Model	AICc	*K*	ΔAICc	Δ_0_AICc	AICcW	Rank
t, sex, MAB	1: *ϕ*_t·MAB²+sex_,*p*_t_	1871.1	24	0	−10.1	0.68	1
	2: *ϕ*_t·MAB²+sex·MAB²_,*p*_t_	1874.5	26	3.4	−6.7	0.12	2
	3: *ϕ*_t+sex+MAB²_,*p*_t_	1875.6	17	4.5	−5.6	0.07	3
	4: *ϕ*_t+sex·MAB²_,*p*_t_	1876.9	18	5.8	−4.3	0.04	4
	5: *ϕ*_t+sex+MAB+MAB²_,*p*_t_	1877.2	18	6.1	−4	0.03	5
	6: *ϕ*_t+sex·(MAB+MAB²)_,*p*_t_	1880.2	20	9.1	−1	0.01	7
t, sex, SB	7: *ϕ*_t+sex+1st(SB²)_,*p*_t_	1878.4	17	7.3	−2.8	0.02	6
	8: *ϕ*_t+sex·1st(SB²)_,*p*_t_	1880.2	18	9.1	−1	0.01	8
	9: *ϕ*_t+sex+1st(SB+SB²)_,*p*_t_	1880.2	18	9.1	−1	0.01	9
t, sex	10: *ϕ*_t+sex_,*p*_t_	1881.2	16	10.1	0	0	10

**Figure 4:**
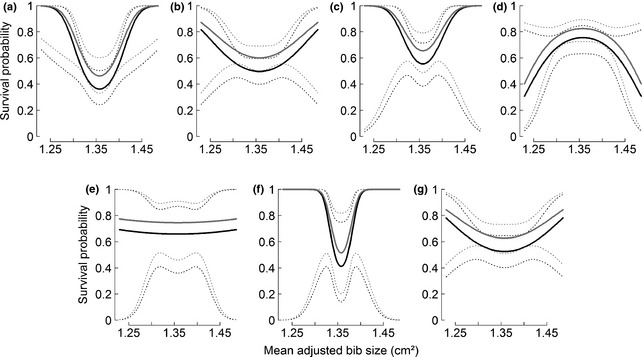
Yearly survival probability according to mean‐adjusted bib size in each time step of the study: (a) 2002–2003, (b) 2003–2004, (c) 2004–2005, (d) 2005–2008, (e) 2008–2009, (f) 2009–2010, (g) 2010–2011. Survival is confounded with recapture probability for the last time step (2011–2012) and thus unidentifiable. The plotted lines represent estimated survival probabilities obtained with the best model (*ϕ*_t·_ _MAB__²+sex_,*p*_t_) which suggests fluctuating viability selection. Solid lines indicate means and dotted lines 95% confidence intervals. Females are plotted in black and males in grey.

Among the five CR models that included MAB, survival was best described by two models that included an interaction between time and a quadratic effect of MAB on long‐term yearly survival (Table 2). Therefore, the best models included some variation among years in the relationship between survival and bib size. The first of these two best models differed from all the other best models by more than 3.4 points of AICc, and from the ‘null model’ by 10.1 points of AICc (Table 2). This model did not include an interaction between sex and bib size. Closer inspection of each year revealed a significant positive quadratic effect (*β*_MAB²_ = 0.5, 95% CI = [0.08,1.08] on the logit scale, Fig. 4) in the first time step (2002–2003), with higher survival for birds possessing small and large bibs. For the remaining six time steps, there was a trend towards a positive quadratic effect in four time steps, a trend towards a negative quadratic effect in one time step and a trend towards a neutral relationship in one time step. Taken together, these results suggest that the U‐shaped relationship between long‐term yearly survival and bib size could be more pronounced in some years than others.

The best model that tested for the effect of SB on short‐term yearly survival included a quadratic effect of bib size on short‐term yearly survival (Table 2), and had an AICc value 2.8 points lower than the ‘null model’. This model did not include an interaction between sex and bib size, but had an AICc value only 1.8 points higher than a very similar model which differed only due to the presence of an interaction between sex and bib size (Table 2), but which was not significant (value of the interaction sex × bib size = 0.24, 95% CI = [–0.07,0.56] on the logit scale). This model showed again that individuals with intermediate bib size had lower survival in the next year than individuals with small and large bibs.

## Discussion

This study investigated the association between a badge of status and survival in a wild population of sociable weavers, and the between‐ and within‐individual variability of this trait. As expected for a signalling trait, we found evidence for high plasticity and variability: the within‐individual variance of bib size was high, and bib size varied between years and was positively correlated to age, body mass and colony size at the population level and/or individual level (although plasticity according to body mass was not significant within individuals). Additionally, we found a clear pattern indicative of disruptive viability selection: both short‐term and long‐term yearly survival showed a U‐shaped relationship with bib size. Birds expressing small and large bibs had better survival than birds with intermediate bib sizes. Last, our results suggested that associations between survival and bib size could fluctuate between years, because the U‐shaped relationship between survival and bib size was more pronounced in some years than others. The pattern of disruptive viability selection found in this study suggests that, as predicted by Maynard Smith and Harper's models (1988, 2003), the social costs associated with bearing a signal of dominance can be strong.

### Survival in relation to bib size

Examples of disruptive viability selection are rare (Calsbeek & Smith, 2008; Bergeron *et al*., 2013) and, to our knowledge, have never been observed for colour signals. Here, we analysed two measures of bib size – MAB and SB – and these appeared to be respectively related to short‐ and long‐term yearly survival (i.e. from 1 year to the next and over the capture–recapture history), in both cases exhibiting a U‐shaped association with survival. Overall, this pattern of disruptive viability selection was both consistent and substantial in the data set: most annual trends linking MAB with survival were towards disruptive viability selection, and this U‐shaped pattern was also significant in the model that did not contain an interaction with time (for both SB and MAB, model 3, 4, 6 and 7, Table 2).

Our results contrast with those obtained by the only two previous CR studies which tested for a quadratic correlation between a colour trait and survival, which found a hump‐shaped relationship and thus suggested stabilizing viability selection. However, these two studies focused on condition‐dependent secondary sexual traits, the carotenoid‐based coloration of beaks (Grégoire *et al*., 2004) and breast feathering (Figuerola & Senar, 2007), whereas here we investigated a melanin‐based trait which instead has primarily a nonsexual, social function (Rat *et al*., 2015).

As described earlier, there are good reasons to predict disruptive viability selection for badges of status. Under the badge‐of‐status hypothesis, individuals having similar badge sizes are expected consistently to interact aggressively, whereas individuals with dissimilar badge sizes are not (Maynard Smith & Harper, 1988, 2003). Being more numerous (Fig. S2), individuals displaying intermediate badge sizes are consequently expected to interact more frequently than others, and this could explain the U‐shaped relationship between bib size and survival if these repeated aggressive interactions are costly. In sociable weavers, bib size is positively associated with social dominance, and medium‐ranked birds engaged more in aggressive interactions than high‐ranked individuals (Rat *et al*., 2015). In group‐living species, however, interactions are often pacified and, in agreement, sociable weavers have frequent agonistic encounters but are seldom engaged in escalated contests, so the costs are likely to be more subtle than injury or death caused by aggressive fights. Costs of agonistic interactions are likely to be physiological and, given the links between androgens or corticosterone and both aggression and eumelanin coloration, there could be long‐term consequences of dominance for oxidative stress or immune function in particular (Creel, 2001; Bókony *et al*., 2008; Ducrest *et al*., 2008; Galván & Alonso‐Alvarez, 2009; Koren *et al*., 2012; Vitousek *et al*., 2013). Costs could also arise from an increase in metabolic rate, as found in some birds and lizards (Senar *et al*., 2000; Buchanan *et al*., 2001; Whiting *et al*., 2003).

Alternatively, the U‐shaped relationship observed between bib size and survival may have different origins, and be only partly linked to the social cost of this signal. For instance, if bib size is a trait of dual utility used in both sexual and social communication, individuals with small bibs could be young individuals that are not currently reproducing, and therefore experience no costs of reproduction and have higher survival probability than breeders. Intermediate and large bib size might in this case reflect, respectively, low‐ and high‐quality/dominant breeders that have, respectively, low and high survival probability. However, as we included age in the statistical models used to estimate the BLUPs, this explanation seems unlikely to account for our results. Moreover, when we removed individuals younger than one year old from the analyses (i.e. individuals we can be sure were not currently reproducing; Covas *et al*., 2004), there was still a trend for disruptive viability selection (the lower significance being partly explained by the reduced sample size, see Appendix S4, Table S5 for details). Hence, the higher survival of individuals with small bibs is unlikely to be related to age.

Although our results clearly indicate a general pattern of disruptive viability selection, MAB analyses suggested that fluctuating viability selection might also occur. Our best capture–recapture model contained an interaction between time and MAB, showing that the U‐shaped relationship between survival and bib size could be more pronounced in some years than others, being only significantly different from zero in 1 year (2002). However, the statistical power, noise or strength of the relationship between survival and MAB did not offer the opportunity to detect significant effects in other years, and prevented precise conclusions about these changes in other years. SB did not suggest any fluctuation in viability selection across years. The different results obtained for the two measures of bib size might reflect lower explanatory power with SB, as this measure involved fewer time steps (5 instead of 8) and fewer individuals at each of these time steps (only the individuals measured at the corresponding occasion) than did the analyses with MAB.

A classical example of fluctuating selection is the beak size of Darwin's finches in the Galapagos (Grant & Grant, 2002). One of the main factors explaining this variation was fluctuation in food availability. Food is also variable in the semi‐desert environment experienced by the sociable weaver and could strongly influence the main costs linked to signalling. We acknowledge that additional years of data are required to verify this fluctuating pattern. Yet, if confirmed, such fluctuating viability selection could arise from variation in survival costs depending on the competitive context influenced by resource availability. For example, as observed in other species, more aggressive interactions might be expected during dry years when food abundance is low, and more peaceful interactions expected when food is abundant (Grant *et al*., 2002; Dubois *et al*., 2003; Rubenstein, 2007). This remains to be tested with more data and potentially experimental manipulations.

Temporal variation in trait optima and selective regimes is interesting because it maintains phenotypic variability within populations (Bell, 2010), potentially explaining why all individuals do not display the same signal. Such variation is expected but rarely investigated (Cornwallis & Uller, 2010). The variation in viability selection found in the present study might constitute a key element explaining the maintenance of the variability in bib size in our study species. However, phenotypic variability in signals is also affected by heritability, degree of assortative mating and reproductive success, and we currently lack information about these mechanisms in sociable weavers.

### Plasticity and variability of bib size

The extent to which the environment alters the expression of melanin signals is debated (Roulin, 2015). Because melanin is endogenously produced and the few studies that quantified heritability in melanin‐based coloration suggested it to be high (with *h* ^*2*^ ranging from 0.53 to 1.0 from five studies on four bird species: Roulin & Ducrest, 2013; but see Chaput‐Bardy *et al*., 2014 who recently found *h²* = 0.18 for wing melanization in a butterfly; and see Griffith *et al*., 1999; Jensen *et al*., 2004), the effect of the environment on melanin‐based traits is sometimes thought to be small. Here, we documented that year strongly affected the variation in bib size. In contrast to the few previous findings mentioned above, this result suggests that the bib size of sociable weavers might be an example of a relatively weakly heritable melanic trait. The fact that sociable weavers live in a highly fluctuating environment with large variation in rainfall could explain the large interannual variation in bib size that we documented. Rainfall greatly affects reproductive success and survival in the study population (Covas *et al*., 2008; Altwegg *et al*., 2014) and therefore is likely to affect investment in signalling through its effect on population density. Rainfall may additionally affect, for instance, the trade‐off between investment in reproduction and ornamentation (e.g. Griffith, 2000; Garant *et al*., 2004; Doutrelant *et al*., 2012; Vergara *et al*., 2012)*,* the level of competition for food (Bretman *et al*., 2011), or even the impact of feather‐degrading bacteria (Burtt & Ichida, 2004). Another environmental factor that might affect the annual level of ornamentation is temperature during moult. Moult has substantial energetic costs (Cyr *et al*., 2008), such that extreme cold temperatures could negatively influence ornament production (Cockburn *et al*., 2008) by increasing the costs of thermoregulation (Gilbert *et al*., 2010).

The second factor associated with variation in bib size was age. Bib size consistently increased with age both within and across individuals. This pattern of age dependency is a common feature in bird ornamentation (e.g. Grant, 1990; Dreiss & Roulin, 2010; Doutrelant *et al*., 2012; Evans & Sheldon, 2013; Potti *et al*., 2013). For both sexual and nonsexual social signals, age dependency can be explained in a life‐history context if signals are costly, and/or it can be explained in a frequency‐dependent context if signal efficiency is relative, depending on the expression of other older and more competitive individuals (Williams, 1966; Kokko, 1997).

Social factors, such as group size and composition, have also been shown to affect signal expression (McGraw *et al*., 2003; Gautier *et al*., 2008; Laucht & Dale, 2012). Interestingly, we found that birds that experienced a change in colony size changed their bib size, producing larger bibs in larger colonies and smaller bibs in smaller colonies (within‐individual effect). In large social groups, there is often more competition for food or mates, leading to an increase in androgen levels, notably testosterone (Adkins‐Regan, 2005; Hill & McGraw, 2006; van Dijk *et al*., 2013) and androstenedione (Gil *et al*., 2007). These may in turn increase the intensity of coloured signals (Adkins‐Regan, 2005; Rubenstein & Hauber, 2008) such as melanin‐based coloration, which is androgen dependent (Bókony *et al*., 2008; Ducrest *et al*., 2008). These links between competition, hormones and coloration could explain the relationship between bib size and colony size. By contrast, there was no effect of colony size on bib size between individuals. This could be explained by the established hierarchy within colonies, each of which contained individuals with large and small bibs independent of its density. Indeed, a recent study showed that sociable weavers are not egalitarian and that their colonies are structured in strongly ordered dominance hierarchies (Rat *et al*., 2015).

Individual condition is also often linked to signal expression, either because the signal is condition dependent or because of pleiotropy, or more simply because any trait has a minimal cost of production and a signal associated with dominance correlates to resource access (Senar, 2006). In agreement, bib size varies with dominance in sociable weavers at both the within‐ and between‐individual levels (Rat *et al*., 2015) and varies with body condition at the between‐individual level (this study). The fact that the bib size of an individual did not increase with its body condition (i.e. no significant within‐individual effect was detected) may stem from the fact that, in our data set, individuals only varied moderately in mass over their lifetimes (repeatability of body mass was high: r = 0.73 ± 0.03, *F*_661,226_ = 4.67, *P* < 0.001). It is possible that we might observe within‐individual variation in bib size if we had the opportunity to manipulate the body condition of sociable weavers. Alternatively, this result might have arisen because bib size is not strongly condition dependent, or because body mass is not a precise estimate of body condition as it was not measured at the time of the moult.

In contrast to studies of sexual dichromatism in some other bird species, we found only a weak sexual difference in the focal trait in our study species. The limited effect of sex on bib size (Fig. S4) suggests that the sexual differentiation of bib size might be practically meaningless. The absence of any difference between the sexes in their associations with the predictor variables influencing bib size (i.e. year, age, body size, colony size) could be explained by a similar function of the ornament in both sexes, and/or by a strong genetic correlation between male and females ornaments (Kraaijeveld *et al*., 2007). The first of these hypotheses is supported by the limited sex differences we found in the relationship between bib size and survival, which arose from a difference in survival between sexes rather than from a difference in the coefficient linking bib size to survival. Indeed, the life‐history traits of sociable weavers would predict that bib size should have similar functions in both sexes: biparental care, absence of promiscuity, high degree of cooperation, high longevity and the absence of migration (Owens, 2006; Kraaijeveld *et al*., 2007; Rubenstein & Lovette, 2009; Doutrelant *et al*., 2013), but this needs to be verified.

## Conclusion

The cost of signals is central to our understanding of social selection (nonsexual, sexual or both). To our knowledge, our study is one of the very few to have tested for a quadratic relationship between ornament expression and survival (Grégoire *et al*., 2004; Figuerola & Senar, 2007), and the first to have documented disruptive viability selection for a badge of status. The pattern of disruptive selection we report suggests that social costs are one of the key factors ensuring the honesty of melanic badges of status in sociable weavers. Although many signals might have more than one function, and/or many social signals may have their honesty ensured by a feedback between social and physiological costs (Tibbetts, 2014), our results call for tests of the direction and shape of the relationship between badge size and survival in other species. Furthermore, our results suggest that large annual variability exists in both bib size expression and its relationship with survival, which now needs to be verified over a longer time series. This fluctuation is interesting because such changes are particularly expected for signals, but this has rarely been documented (only ten species were inventoried by Svensson & Gosden, 2007). In addition, this temporal variation fits well with the current view that signals have multiple functions and costs (Tibbetts, 2014) and that both sexual and nonsexual components of social selection are important in understanding signal evolution (Lyon & Montgomerie, 2012).

## Supporting information


**Appendix S1** Details on bib size measurements.
**Appendix S2** Details on the within‐individual centering approach.
**Appendix S3** Details on the selection of models describing bib size variability.
**Appendix S4** Details on the models describing survival according to bib size.
**Table S1** ANOVA examining the overall effects on bib size of photographer identity and year (nested within photographer identity).
**Table S2** Posthoc Tukey's HSD test following the ANOVA examining the overall effects on bib size of photographer identity and year.
**Table S3** Details on model selection for the study of bib size variability with AICc and R² values.
**Table S4** Summary of the main set of capture‐recapture models.
**Table S5** Summary of the additional set of capture‐recapture models (without individuals younger than one year old).
**Figure S1** Examples of photos used to take bib size measurements.
**Figure S2** Distribution of bib size within each year and pooled across all years.
**Figure S3** Survival probability according to mean‐adjusted bib size in each time step of the study under the best model without interaction between time and bib size (*ϕ*_t+MAB²+sex_,*p*_t_).
**Figure S4** Distribution of bib size in males and females.

## References

[jeb12717-bib-0001] Adkins‐Regan, E. 2005 Hormones and Animal Social Behavior. Princeton University Press, New Jersey.

[jeb12717-bib-0002] Altwegg, R. , Doutrelant, C. , Anderson, M.D. , Spottiswoode, C.N. & Covas, R. 2014 Climate, social factors and research disturbance influence population dynamics in a declining sociable weaver metapopulation. Oecologia 174: 413–425. 24072437 10.1007/s00442-013-2768-7

[jeb12717-bib-0003] Andersson, M. 1994 Sexual Selection. Princeton University Press, Princeton.

[jeb12717-bib-0004] Basolo, A.L. & Wagner, W.E. 2004 Covariation between predation risk, body size and fin elaboration in the green swordtail, *Xiphophorus helleri* . Biol. J. Linn. Soc. 83: 87–100.

[jeb12717-bib-0005] Bell, G. 2010 Fluctuating selection: the perpetual renewal of adaptation in variable environments. Philos. Trans. R. Soc. Lond., B, Biol. Sci. 365: 87–97. 20008388 10.1098/rstb.2009.0150 PMC2842698

[jeb12717-bib-0006] Bergeron, P. , Montiglio, P.‐O. , Réale, D. , Humphries, M.M. , Gimenez, O. & Garant, D. 2013 Disruptive viability selection on adult exploratory behaviour in eastern chipmunks. J. Evol. Biol. 26: 766–774. 23437956 10.1111/jeb.12081

[jeb12717-bib-0007] Berglund, A. , Bisazza, A. & Pilastro, A. 1996 Armaments and ornaments: an evolutionary explanation of traits of dual utility. Biol. J. Linn. Soc. 58: 385–399.

[jeb12717-bib-0008] Bergman, T.J. , Ho, L. & Beehner, J.C. 2009 Chest color and social status in male geladas (*Theropithecus gelada*). Int. J. Primatol. 30: 791–806.

[jeb12717-bib-0009] Bize, P. , Gasparini, J. , Klopfenstein, A. , Altwegg, R. & Roulin, A. 2006 Melanin‐based coloration is a nondirectionally selected sex‐specific signal of offspring development in the alpine swift. Evolution 60: 2370–2380. 17236427

[jeb12717-bib-0010] Bókony, V. , Garamszegi, L.Z. , Hirschenhauser, K. & Liker, A. 2008 Testosterone and melanin‐based black plumage coloration: a comparative study. Behav. Ecol. Sociobiol. 62: 1229–1238.

[jeb12717-bib-0011] Bolker, B.M. , Brooks, M.E. , Clark, C.J. , Geange, S.W. , Poulsen, J.R. , Stevens, M.H.H. *et al* 2009 Generalized linear mixed models: a practical guide for ecology and evolution. Trends Ecol. Evol. 24: 127–135. 19185386 10.1016/j.tree.2008.10.008

[jeb12717-bib-0012] Bretman, A. , Gage, M.J.G. & Chapman, T. 2011 Quick‐change artists: male plastic behavioural responses to rivals. Trends Ecol. Evol. 26: 467–473. 21680050 10.1016/j.tree.2011.05.002

[jeb12717-bib-0013] Bro‐Jørgensen, J. & Beeston, J. 2015 Multimodal signalling in an antelope: fluctuating facemasks and knee‐clicks reveal the social status of eland bulls. Anim. Behav. 102: 231–239.

[jeb12717-bib-0014] Brommer, J.E. , Wilson, A.J. & Gustafsson, L. 2007 Exploring the genetics of aging in a wild passerine bird. Am. Nat. 170: 643–650. 17891742 10.1086/521241

[jeb12717-bib-0015] Buchanan, K.L. , Evans, M.R. , Goldsmith, A.R. , Bryant, D.M. & Rowe, L. 2001 Testosterone influences basal metabolic rate in male house sparrows: a new cost of dominance signalling? Proc. Biol. Sci. 268: 1337–1344. 11429132 10.1098/rspb.2001.1669 PMC1088746

[jeb12717-bib-0016] Burnham, K.P. & Anderson, D.R. 1998 Model selection and inference: a practical information‐theoretic approach. Springer, New York.

[jeb12717-bib-0017] Burtt, E.H. & Ichida, J.M. 2004 Gloger's rule, feather‐degrading bacteria, and color variation among Song Sparrows. Condor 106: 681–686.

[jeb12717-bib-0018] Calsbeek, R. & Smith, T.B. 2008 Experimentally replicated disruptive selection on performance traits in a Caribbean lizard. Evolution 62: 478–484. 18053075 10.1111/j.1558-5646.2007.00282.x

[jeb12717-bib-0019] Chaput‐Bardy, A. , Ducatez, S. , Legrand, D. & Baguette, M. 2014 Fitness costs of thermal reaction norms for wing melanisation in the large white butterfly (*Pieris brassicae*). PLoS ONE 9: e90026. 24587196 10.1371/journal.pone.0090026 PMC3937413

[jeb12717-bib-0020] Choquet, R. , Rouan, L. & Pradel, R. 2009a Program E‐SURGE: a software for fitting multievent models In: Modeling Demographic Processes in Marked Populations (Thomson D.L., Cooch E.G., Conroy M.J., eds), pp. 847–868. Springer, Berlin.

[jeb12717-bib-0021] Choquet, R. , Lebreton, J.‐D. , Gimenez, O. , Reboulet, A.‐M. & Pradel, R. 2009b U‐CARE: Utilities for performing goodness of fit tests and manipulating capture‐recapture data. Ecography 32: 1071–1074.

[jeb12717-bib-0022] Cockburn, A. , Osmond, H.L. & Double, M.C. 2008 Swingin' in the rain: condition dependence and sexual selection in a capricious world. Proc. Biol. Sci. 275: 605–612. 18211882 10.1098/rspb.2007.0916 PMC2596836

[jeb12717-bib-0023] Cornwallis, C.K. & Uller, T. 2010 Towards an evolutionary ecology of sexual traits. Trends Ecol. Evol. 25: 145–152. 19853321 10.1016/j.tree.2009.09.008

[jeb12717-bib-0024] Covas, R. , Doutrelant, C. & du Plessis, M.A. 2004 Experimental evidence of a link between breeding conditions and the decision to breed or to help in a colonial cooperative bird. Proc. Biol. Sci. 271: 827–832. 15255101 10.1098/rspb.2003.2652 PMC1691664

[jeb12717-bib-0025] Covas, R. , du Plessis, M.A. & Doutrelant, C. 2008 Helpers in colonial cooperatively breeding sociable weavers *Philetairus socius* contribute to buffer the effects of adverse breeding conditions. Behav. Ecol. Sociobiol. 63: 103–112.

[jeb12717-bib-0026] Covas, R. , Deville, A.‐S. , Doutrelant, C. , Spottiswoode, C.N. & Grégoire, A. 2011 The effect of helpers on the postfledging period in a cooperatively breeding bird, the sociable weaver. Anim. Behav. 81: 121–126.

[jeb12717-bib-0027] Creel, S. 2001 Social dominance and stress hormones. Trends Ecol. Evol. 16: 491–497.

[jeb12717-bib-0028] Cyr, N.E. , Wikelski, M. & Romero, L.M. 2008 Increased energy expenditure but decreased stress responsiveness during molt. Physiol. Biochem. Zool. 81: 452–462. 18537472 10.1086/589547

[jeb12717-bib-0029] Dey, C.J. , Dale, J. & Quinn, J.S. 2014 Manipulating the appearance of a badge of status causes changes in true badge expression. Proc. Biol. Sci. 281: 20132680. 24285201 10.1098/rspb.2013.2680 PMC3866412

[jeb12717-bib-0030] van Dijk, R.E. , Eising, C.M. , Merrill, R.M. , Karadas, F. , Hatchwell, B.J. & Spottiswoode, C.N. 2013 Maternal effects in the highly communal sociable weaver may exacerbate brood reduction and prepare offspring for a competitive social environment. Oecologia 173: 379–389. 22948278 10.1007/s00442-012-2439-0

[jeb12717-bib-0031] Doutrelant, C. , Grégoire, A. , Midamegbe, A. , Lambrechts, M. & Perret, P. 2012 Female plumage coloration is sensitive to the cost of reproduction. An experiment in blue tits. J. Anim. Ecol. 81: 87–96. 21819397 10.1111/j.1365-2656.2011.01889.x

[jeb12717-bib-0032] Doutrelant, C. , Grégoire, A. , Gomez, D. , Staszewski, V. , Arnoux, E. , Tveraa, T. *et al* 2013 Colouration in Atlantic puffins and blacklegged kittiwakes: monochromatism and links to body condition in both sexes. J. Avian Biol. 44: 451–460.

[jeb12717-bib-0033] Dreiss, A.N. & Roulin, A. 2010 Age‐related change in melanin‐based coloration of Barn owls (*Tyto alba*): females that become more female‐like and males that become more male‐like perform better. Biol. J. Linn. Soc. 101: 689–704.

[jeb12717-bib-0034] Dubois, F. , Giraldeau, L. & Grant, J. 2003 Resource defense in a group‐foraging context. Behav. Ecol. 14: 2–9.

[jeb12717-bib-0035] Ducrest, A.‐L. , Keller, L. & Roulin, A. 2008 Pleiotropy in the melanocortin system, coloration and behavioural syndromes. Trends Ecol. Evol. 23: 502–510. 18644658 10.1016/j.tree.2008.06.001

[jeb12717-bib-0036] Emaresi, G. , Bize, P. , Altwegg, R. , Henry, I. , van den Brink, V. , Gasparini, J. *et al* 2014 Melanin‐specific life‐history strategies. Am. Nat. 183: 269–280. 24464200 10.1086/674444

[jeb12717-bib-0037] Evans, S.R. & Sheldon, B.C. 2013 Pigments versus structure: examining the mechanism of age‐dependent change in a carotenoid‐based colour. J. Anim. Ecol. 82: 418–428. 23194384 10.1111/1365-2656.12008

[jeb12717-bib-0038] Figuerola, J. & Senar, J.C. 2007 Serins with intermediate brightness have a higher survival in the wild. Oikos 116: 636–641.

[jeb12717-bib-0039] Fox, D.L. 1976 Animal biochromes and structural colors. University of California Press, Berkeley.

[jeb12717-bib-0040] Galván, I. & Alonso‐Alvarez, C. 2009 The expression of melanin‐based plumage is separately modulated by exogenous oxidative stress and a melanocortin. Proc. Biol. Sci. 276: 3089–3097. 19520801 10.1098/rspb.2009.0774 PMC2817136

[jeb12717-bib-0041] Garant, D. , Sheldon, B.C. & Gustafsson, L. 2004 Climatic and temporal effects on the expression of secondary sexual characters: genetic and environmental components. Evolution 58: 634–644. 15119446

[jeb12717-bib-0042] Garcia‐Berthou, E. 2001 On the misuse of residuals in ecology: testing regression residuals vs. the analysis of covariance. J. Anim. Ecol. 70: 708–711.

[jeb12717-bib-0043] Gautier, P. , Barroca, M. , Bertrand, S. , Eraud, C. , Gaillard, M. , Hamman, M. *et al* 2008 The presence of females modulates the expression of a carotenoid‐based sexual signal. Behav. Ecol. Sociobiol. 62: 1159–1166.

[jeb12717-bib-0044] Gil, D. , Biard, C. , Lacroix, A. , Spottiswoode, C.N. , Saino, N. , Puerta, M. *et al* 2007 Evolution of yolk androgens in birds: development, coloniality, and sexual dichromatism. Am. Nat. 169: 802–819. 17479466 10.1086/516652

[jeb12717-bib-0045] Gilbert, C. , McCafferty, D. , Le Maho, Y. , Martrette, J.‐M. , Giroud, S. , Blanc, S. *et al* 2010 One for all and all for one: the energetic benefits of huddling in endotherms. Biol. Rev. 85: 545–569. 20039866 10.1111/j.1469-185X.2009.00115.x

[jeb12717-bib-0046] Gimenez, O. , Viallefont, A. , Charmantier, A. , Pradel, R. , Cam, E. , Brown, C. *et al* 2008 The risk of flawed inference in evolutionary studies when detectability is less than one. Am. Nat. 172: 441–448. 18657010 10.1086/589520

[jeb12717-bib-0047] Goss, R.J. 2012 Deer antlers: regeneration, function and evolution. Academic Press, London.

[jeb12717-bib-0048] Grafen, A. 1990 Sexual selection unhandicapped by the Fisher process. J. Theor. Biol. 144: 473–516. 2402152 10.1016/s0022-5193(05)80087-6

[jeb12717-bib-0049] Grant, B.R. 1990 The significance of subadult plumage in Darwin's finches, *Geospiza fortis* . Behav. Ecol. 1: 161–170.

[jeb12717-bib-0050] Grant, P.R. & Grant, B.R. 2002 Unpredictable evolution in a 30‐year study of Darwin's finches. Science 296: 707–711. 11976447 10.1126/science.1070315

[jeb12717-bib-0051] Grant, J.W. , Girard, I.L. , Breau, C. & Weir, L.K. 2002 Influence of food abundance on competitive aggression in juvenile convict cichlids. Anim. Behav. 63: 323–330.

[jeb12717-bib-0052] Grégoire, A. , Preault, M. , Cezilly, F. , Wood, M.J. , Pradel, R. & Faivre, B. 2004 Stabilizing natural selection on the early expression of a secondary sexual trait in a passerine bird. J. Evol. Biol. 17: 1152–1156. 15312087 10.1111/j.1420-9101.2004.00756.x

[jeb12717-bib-0053] Griffith, S.C. 2000 A trade‐off between reproduction and a condition‐dependent sexually selected ornament in the house sparrow *Passer domesticus* . Proc. Biol. Sci. 267: 1115–1119. 10885516 10.1098/rspb.2000.1116 PMC1690642

[jeb12717-bib-0054] Griffith, S.C. , Owens, I.P.F. & Burke, T. 1999 Environmental determination of a sexually selected trait. Nature 400: 358–360.

[jeb12717-bib-0055] Griffith, S.C. , Parker, T.H. & Olson, V.A. 2006 Melanin‐ versus carotenoid‐based sexual signals: is the difference really so black and red? Anim. Behav. 71: 749–763.

[jeb12717-bib-0056] Griffiths, R. , Double, M.C. , Orr, K. & Dawson, R.J.G. 1998 A DNA test to sex most birds. Mol. Ecol. 7: 1071–1075. 9711866 10.1046/j.1365-294x.1998.00389.x

[jeb12717-bib-0057] Hill, G.E. 1993 Male mate choice and the evolution of female plumage coloration in the house finch. Evolution 47: 1515–1525. 10.1111/j.1558-5646.1993.tb02172.x 28564892

[jeb12717-bib-0058] Hill, G.E. & McGraw, K.J. 2006 Bird coloration, vol. 2. Function and evolution. Harvard University Press, Cambridge, Function and evolution.

[jeb12717-bib-0059] Hunt, J. , Brooks, R.C. , Jennions, M.D. , Smith, M.J. , Bentsen, C.L. & Bussière, L.F. 2004 High‐quality male field crickets invest heavily in sexual display but die young. Nature 432: 1024–1027. 15616562 10.1038/nature03084

[jeb12717-bib-0060] Jennions, M. , Møller, A. & Petrie, M. 2001 Sexually selected traits and adult survival: a meta‐analysis. Q. Rev. Biol. 76: 3–36. 11291569 10.1086/393743

[jeb12717-bib-0061] Jensen, H. , Saether, B.E. , Ringsby, T.H. , Tufto, J. , Griffith, S.C. & Ellegren, H. 2004 Lifetime reproductive success in relation to morphology in the house sparrow *Passer domesticus* . J. Anim. Ecol. 73: 599–611.

[jeb12717-bib-0062] Johnson, A.M. & Fuller, R.C. 2015 The meaning of melanin, carotenoid, and pterin pigments in the bluefin killifish, *Lucania goodei* . Behav. Ecol. 26: 158–167.

[jeb12717-bib-0063] Johnson, J.B. & Omland, K.S. 2004 Model selection in ecology and evolution. Trends Ecol. Evol. 19: 101–108. 16701236 10.1016/j.tree.2003.10.013

[jeb12717-bib-0064] Jones, I.L. , Hunter, F. , Robertson, G. & Fraser, G. 2004 Natural variation in the sexually selected feather ornaments of crested auklets (*Aethia cristatella*) does not predict future survival. Behav. Ecol. 15: 332–337.

[jeb12717-bib-0065] Kodric‐Brown, A. 1998 Sexual dichromatism and temporary color changes in the reproduction of fishes. Am. Zool. 38: 70–81.

[jeb12717-bib-0066] Kokko, H. 1997 Evolutionarily stable strategies of age‐dependent sexual advertisement. Behav. Ecol. Sociobiol. 41: 99–107.

[jeb12717-bib-0067] Koren, L. , Nakagawa, S. , Burke, T. , Soma, K.K. , Wynne‐Edwards, K.E. & Geffen, E.E. 2012 Non‐breeding feather concentrations of testosterone, corticosterone and cortisol are associated with subsequent survival in wild house sparrows. Proc. Biol. Sci. 279: 1560–1566. 22090380 10.1098/rspb.2011.2062 PMC3282351

[jeb12717-bib-0068] Kraaijeveld, K. , Kraaijeveld‐Smit, F.J.L. & Komdeur, J. 2007 The evolution of mutual ornamentation. Anim. Behav. 74: 657–677.

[jeb12717-bib-0069] Laucht, S. & Dale, J. 2012 Development of badges of status in captive male house sparrows (*Passer domesticus*) in relation to the relative ornamentation of flock‐mates. Ethology 118: 644–653.

[jeb12717-bib-0070] Lebreton, J.‐D. , Burnham, K.P. , Clobert, J. & Anderson, D.R. 1992 Modeling survival and testing biological hypotheses using marked animals: a unified approach with case studies. Ecol. Monogr. 62: 67–118.

[jeb12717-bib-0071] Lyon, B.E. & Montgomerie, R. 2012 Sexual selection is a form of social selection. Philos. Trans. R. Soc. Lond., B, Biol. Sci. 367: 2266–2273. 22777015 10.1098/rstb.2012.0012 PMC3391428

[jeb12717-bib-0072] Maclean, G.L. 1973 The sociable weaver. Ostrich 44: 176–261.

[jeb12717-bib-0073] Mäthger, L.M. , Denton, E.J. , Marshall, N.J. & Hanlon, R.T. 2009 Mechanisms and behavioural functions of structural coloration in cephalopods. J. R. Soc. Interface 6: S149–S163. 19091688 10.1098/rsif.2008.0366.focus PMC2706477

[jeb12717-bib-0074] Maynard Smith, J. & Harper, D. 1988 The evolution of aggression: can selection generate variability? Philos. Trans. R. Soc. Lond., B, Biol. Sci. 319: 557–570. 2905492 10.1098/rstb.1988.0065

[jeb12717-bib-0075] Maynard Smith, J. & Harper, D. 2003 Animal Signals. Oxford University Press, Oxford.

[jeb12717-bib-0076] McCullough, E.L. & Emlen, D.J. 2013 Evaluating the costs of a sexually selected weapon: big horns at a small price. Anim. Behav. 86: 977–985.

[jeb12717-bib-0077] McGraw, K.J. 2006 Mechanics of melanin‐based coloration In: Bird Coloration Vol 1. Mechanisms and Measurements (Hill G.E., McGraw K.J., eds), pp. 243–294. Harvard University Press, Cambridge.

[jeb12717-bib-0078] McGraw, K.J. 2008 An update on the honesty of melanin‐based color signals in birds. Pigment Cell Melanoma Res. 21: 133–138. 18426406 10.1111/j.1755-148X.2008.00454.x

[jeb12717-bib-0079] McGraw, K.J. , Dale, J. & Mackillop, E.A. 2003 Social environment during molt and the expression of melanin‐based plumage pigmentation in male house sparrows (*Passer domesticus*). Behav. Ecol. Sociobiol. 53: 116–122.

[jeb12717-bib-0080] Meunier, J. , Figueiredo Pinto, S. , Burri, R. & Roulin, A. 2011 Eumelanin‐based coloration and fitness parameters in birds: a meta‐analysis. Behav. Ecol. Sociobiol. 65: 559–567.

[jeb12717-bib-0081] Nakagawa, S. & Schielzeth, H. 2010 Repeatability for Gaussian and non‐Gaussian data: a practical guide for biologists. Biol. Rev. 85: 935–956. 20569253 10.1111/j.1469-185X.2010.00141.x

[jeb12717-bib-0082] Nakagawa, S. & Schielzeth, H. 2013 A general and simple method for obtaining R² from generalized linear mixed‐effects models. Methods Ecol. Evol. 4: 133–142.

[jeb12717-bib-0083] Nilsson Sköld, H. , Aspengren, S. & Wallin, M. 2013 Rapid color change in fish and amphibians–function, regulation, and emerging applications. Pigment Cell Melanoma Res. 26: 29–38. 23082932 10.1111/pcmr.12040

[jeb12717-bib-0084] Osborne, L. 2005 Information content of male agonistic displays in the territorial tawny dragon (*Ctenophorus decresii*). J. Ethol. 23: 189–197.

[jeb12717-bib-0085] Owens, I.P.F. 2006 Ecological explanations for interspecific variability in coloration In: Bird Coloration Vol. 2 Function and Evolution (Hill G.E., McGraw K.J., eds), pp. 380–416. Harvard University Press, Cambridge.

[jeb12717-bib-0086] Pinheiro, J.C. & Bates, D.M. 2000 Mixed‐Effects Models in S and S‐PLUS. Springer, New York.

[jeb12717-bib-0087] van de Pol, M. & Wright, J. 2009 A simple method for distinguishing within‐ versus between‐subject effects using mixed models. Anim. Behav. 77: 753–758.

[jeb12717-bib-0088] Potti, J. , Canal, D. & Serrano, D. 2013 Lifetime fitness and age‐related female ornament signalling: evidence for survival and fecundity selection in the pied flycatcher. J. Evol. Biol. 26: 1445–1457. 23638705 10.1111/jeb.12145

[jeb12717-bib-0089] Preston, B.T. , Saint Jalme, M. , Hingrat, Y. , Lacroix, F. & Sorci, G. 2011 Sexually extravagant males age more rapidly. Ecol. Lett. 14: 1017–1024. 21806745 10.1111/j.1461-0248.2011.01668.x

[jeb12717-bib-0090] Qvarnström, A. & Forsgren, E. 1998 Should females prefer dominant males?. Trends Ecol. Evol. 13: 498–501. 21238407 10.1016/s0169-5347(98)01513-4

[jeb12717-bib-0091] R Core Team 2012 R: A Language and Environment for Statistical Computing. R Foundation for Statistical Computing, Vienna, Austria http://www.R-project.org/.

[jeb12717-bib-0092] Rat, M. , van Dijk, R.E. , Covas, R. & Doutrelant, C. 2015 Dominance hierarchies and associated signalling in a cooperative passerine. Behav. Ecol. Sociobiol. 69: 437–448.

[jeb12717-bib-0093] Roff, D.A. & Fairbairn, D.J. 2013 The costs of being dark: the genetic basis of melanism and its association with fitness‐related traits in the sand cricket. J. Evol. Biol. 26: 1406–1416. 23675858 10.1111/jeb.12150

[jeb12717-bib-0094] Rohwer, S. 1977 Status signaling in Harris sparrows : some experiments in deception. Behaviour 61: 107–129.

[jeb12717-bib-0095] Roulin, A. 2015 Condition‐dependence, pleiotropy and the handicap principle of sexual selection in melanin‐based colouration. Biol. Rev. doi: 10.1111/brv.12171. 10.1111/brv.12171 25631160

[jeb12717-bib-0096] Roulin, A. & Altwegg, R. 2007 Breeding rate is associated with pheomelanism in male and with eumelanism in female barn owls. Behav. Ecol. 18: 563–570.

[jeb12717-bib-0097] Roulin, A. & Ducrest, A.‐L. 2013 Genetics of colouration in birds. Semin. Cell Dev. Biol. 24: 594–608. 23665152 10.1016/j.semcdb.2013.05.005

[jeb12717-bib-0098] Rubenstein, D.R. 2007 Stress hormones and sociality: integrating social and environmental stressors. Proc. Biol. Sci. 274: 967–975. 17251100 10.1098/rspb.2006.0051 PMC2141667

[jeb12717-bib-0099] Rubenstein, D.R. & Hauber, M.E. 2008 Dynamic feedback between phenotype and physiology in sexually selected traits. Trends Ecol. Evol. 23: 655–658. 18951654 10.1016/j.tree.2008.07.010

[jeb12717-bib-0100] Rubenstein, D.R. & Lovette, I.J. 2009 Reproductive skew and selection on female ornamentation in social species. Nature 462: 786–789. 20010686 10.1038/nature08614

[jeb12717-bib-0101] Safran, R.J. , Adelman, J.S. , McGraw, K.J. & Hau, M. 2008 Sexual signal exaggeration affects physiological state in male barn swallows. Curr. Biol. 18: R461–R462. 18522812 10.1016/j.cub.2008.03.031

[jeb12717-bib-0102] Searcy, W.A. & Nowicki, S. 2005 The evolution of animal communication. Princeton University Press, Princeton.

[jeb12717-bib-0103] Senar, J.C. 2006 Color displays as intrasexual signals of aggression and dominance In: Bird Coloration Vol. 2. Function and Evolution (Hill G.E., McGraw K.J., eds), pp. 87–136. Harvard University Press, Oxford.

[jeb12717-bib-0104] Senar, J.C. , Polo, V. , Uribe, F. & Camerino, M. 2000 Status signalling, metabolic rate and body mass in the siskin: the cost of being a subordinate. Anim. Behav. 59: 103–110. 10640372 10.1006/anbe.1999.1281

[jeb12717-bib-0105] Stoehr, A.M. 2006 Costly melanin ornaments: the importance of taxon? Funct. Ecol. 20: 276–281.

[jeb12717-bib-0106] Stuart‐Fox, D.M. , Moussalli, A. , Marshall, N.J. & Owens, I.P.F. 2003 Conspicuous males suffer higher predation risk: visual modelling and experimental evidence from lizards. Anim. Behav. 66: 541–550.

[jeb12717-bib-0107] Svensson, E.I. & Gosden, T.P. 2007 Contemporary evolution of secondary sexual traits in the wild. Funct. Ecol. 21: 422–433.

[jeb12717-bib-0108] Tibbetts, E.A. 2014 The evolution of honest communication: integrating social and physiological costs of ornamentation. Integr. Comp. Biol. 54: 578–590. 24944118 10.1093/icb/icu083

[jeb12717-bib-0109] Tibbetts, E.A. & Dale, J. 2004 A socially enforced signal of quality in a paper wasp. Nature 432: 218–222. 15538369 10.1038/nature02949

[jeb12717-bib-0110] Tibbetts, E.A. & Safran, R.J. 2009 Co‐evolution of plumage characteristics and winter sociality in New and Old World sparrows. J. Evol. Biol. 22: 2376–2386. 19874438 10.1111/j.1420-9101.2009.01861.x

[jeb12717-bib-0111] Toms, J.D. & Lesperance, M.L. 2003 Piecewise regression: a tool for identifying ecological thresholds. Ecology 84: 2034–2041.

[jeb12717-bib-0112] Vergara, P. , Redpath, S.M. , Martínez‐Padilla, J. & Mougeot, F. 2012 Environmental conditions influence red grouse ornamentation at a population level. Biol. J. Linn. Soc. 107: 788–798.

[jeb12717-bib-0113] Vitousek, M.N. , Stewart, R.A. & Safran, R.J. 2013 Female plumage colour influences seasonal oxidative damage and testosterone profiles in a songbird. Biol. Lett. 9: 20130539. 23966597 10.1098/rsbl.2013.0539 PMC3971695

[jeb12717-bib-0114] Whiting, M.J. , Nagy, K.A. & Bateman, P.W. 2003 Evolution and maintenance of social status signalling badges: experimental manipulations in lizards In: Lizard Social Behavior (Fox S.F., McCoy J.K., Baird T.A., eds), pp. 47–82. Johns Hopkins University Press, Baltimore.

[jeb12717-bib-0115] Williams, G.C. 1966 Natural selection, the costs of reproduction, and a refinement of Lack's principle. Am. Nat. 100: 687–690.

[jeb12717-bib-0116] Zahavi, A. 1975 Mate selection ‐ a selection for a handicap. J. Theor. Biol. 53: 205–214. 1195756 10.1016/0022-5193(75)90111-3

