# Supplementary material for: Disruptive viability selection on a black plumage trait associated with dominance
Source: J Evol Biol. 2015 Sep 14;28(11):2027–41. doi: 10.1111/jeb.12717 (PMC4949555; doi:10.1111/jeb.12717)
Supplement: Supplementary file 1 — Appendix S1 Details on bib size measurements. Appendix S2 Details on the within‐individual centering approach. Appendix S3 Details on the selection of models describing bib size variability. Appendix S4 Details on the models describing survival according to bib size. Table S1 ANOVA examining the overall effects on bib size of photographer identity and year (nested within photographer identity). Table S2 Posthoc Tukey's HSD test following the ANOVA examining the overall effects on bib size of photographer identity and year. Table S3 Details on model selection for the study of bib size variability with AICc and R² values. Table S4 Summary of the main set of capture‐recapture models. Table S5 Summary of the additional set of capture‐recapture models (without individuals younger than one year old). Figure S1 Examples of photos used to take bib size measurements. Figure S2 Distribution of bib size within each year and pooled across all years. Figure S3 Survival probability according to mean‐adjusted bib size in each time step of the study under the best model without interaction between time and bib size (ϕ t+MAB²+sex,p t). Figure S4 Distribution of bib size in males and females. [file JEB-28-2027-s001.docx]

**Supporting Information**

**Disruptive viability selection on a black plumage trait associated with dominance**

**Paul Acker ^a,b*^, Arnaud Grégoire^a^, Margaux Rat**^c^**, Claire N. Spottiswoode**^c,d^**, René E. van Dijk^e^, Matthieu Paquet^a,c^, Jennifer C. Kaden**^e,f^**, Roger Pradel^a^, Ben J. Hatchwell**^e^**, Rita Covas**^c,g,h**^**, Claire Doutrelant ^a,c**^**

^a^ CEFE UMR 5175, CNRS - Université de Montpellier - Université Paul-Valéry Montpellier - EPHE, 1919 route de Mende, F-34293 Montpellier, Cedex 05 France.

^b^ Université Toulouse 3 Paul Sabatier, CNRS, ENFA ; UMR 5174 EDB (Laboratoire Évolution & Diversité Biologique) ; 118 route de Narbonne, F-31062 Toulouse, France.

^c^ Percy FitzPatrick Institute, DST/NRF Centre of Excellence, University of Cape Town, Rondebosch 7701, South Africa.

^d^ Department of Zoology, University of Cambridge, Downing Street, Cambridge, CB2 3EJ, United Kingdom.

^e^ Department of Animal and Plant Sciences, University of Sheffield, Western Bank, Sheffield S10 2TN, United Kingdom.

^f^ The Royal Zoological Society of Scotland Edinburgh Zoo 134 Corstorphine Road, Edinburgh, EH12 6TS, United Kingdom.

^g^ CIBIO, University of Porto, Rua Monte-Crasto, 4485-661 Vairão, Portugal.

^h^ Biology Department, Science Faculty, University of Porto, Porto, Portugal.

***** Corresponding author: paul.acker@univ-tlse3.fr, +33 5 61 55 67 56

** These two authors contributed equally to the work

**Appendix S1: Bib size measurements**

To take measurements of bib size we processed the photos taken in the field (Fig. S1) using Adobe Photoshop CS6. We selected all the black plumage around the beak and measured the number of pixels it contained. We used the ruler to scale photos and count the number of pixels in 1 cm². With this method we obtained measures of the bib area in cm².

We were confident that there was no effect of the photographer on bib size because all used the same commonly used and robust method to quantify patch size, and all photographers held the birds in a standardised way. This was confirmed by an ANOVA of bib size explained by year nested within photographer identity, which showed that years were significantly different within photographers (*P* < 0.001, Table S1), and for each photographer there was at least one year of photographs that was not significantly different from at least one year of photographs taken by another photographer (TukeyHSD test: *P* > 0.34 for 4 pairs of years and photographers, Table S2).

Result summaries are given hereafter: (i) the ANOVA table (Table S1), (ii) the results of a Tukey's HSD (Honest Significant Difference) test that compares means between each nested subgroup (i.e. years within photographers, Table S2).

**Table S1.** Results of a nested ANOVA examining the overall effects on bib size of photographer identity (MR, CS, RVD) and year (2002-2004,2010-2012, nested within photographer identity).

|  | d.f. | Sum of squares | Mean squares | F | *P*-value |
| --- | --- | --- | --- | --- | --- |
| Photographer | 2 | 7.64 | 3.820 | 103.41 | < 0.001 |
| Photographer:Year | 3 | 3.82 | 1.272 | 34.25 | < 0.001 |
| Residuals | 882 | 32.58 | 0.037 |  |  |

**Table S2.** Posthoc Tukey's Honest Significant Difference test for pairwise comparisons performed in conjunction of the nested ANOVA examining the effects on bib size of photographer identity (MR, CS, RVD) and year (2002-2004,2010-2012, nested within photographer identity).

|  | Difference | 95% confidence interval | | *P*-value |
| --- | --- | --- | --- | --- |
|  |  | lower | upper |  |
| Photographer |  |  |  |  |
| MR - CS | -0.192 | -0.225 | -0.159 | < 0.001 |
| RVD - CS | -0.172 | -0.216 | -0.127 | < 0.001 |
| RVD - MR | 0.020 | -0.025 | 0.065 | 0.547 |
|  |  |  |  |  |
| Photographer:Year |  |  |  |  |
| CS:2003-CS:2002 | 0.205 | 0.129 | 0.281 | < 0.001 |
| CS:2004-CS:2002 | 0.221 | 0.117 | 0.325 | < 0.001 |
| RVD:2010-CS:2002 | -0.029 | -0.112 | 0.053 | < 0.001 |
| MR:2011-CS:2002 | -0.039 | -0.114 | 0.036 | 0.935 |
| MR:2012-CS:2002 | -0.066 | -0.149 | 0.018 | 0.341 |
| CS:2004-CS:2003 | 0.015 | -0.082 | 0.113 | > 0.999 |
| RVD:2010-CS:2003 | -0.235 | -0.308 | -0.161 | < 0.001 |
| MR:2011-CS:2003 | -0.245 | -0.310 | -0.180 | < 0.001 |
| MR:2012-CS:2003 | -0.271 | -0.346 | -0.197 | < 0.001 |
| RVD:2010-CS:2004 | -0.250 | -0.353 | -0.147 | < 0.001 |
| MR:2011-CS:2004 | -0.260 | -0.357 | -0.163 | < 0.001 |
| MR:2012-CS:2004 | -0.287 | -0.390 | -0.183 | < 0.001 |
| MR:2011-RVD:2010 | -0.010 | -0.083 | 0.063 | 1.000 |
| MR:2012-RVD:2010 | -0.037 | -0.118 | 0.045 | 0.984 |
| MR:2012-MR:2011 | -0.026 | -0.100 | 0.047 | 0.999 |


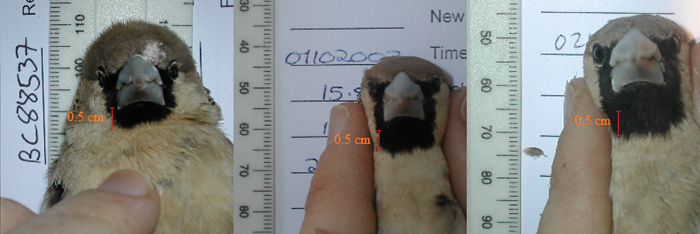


**Figure S1**. Examples of photos used to take bib size measurements. From left to right: an adult with a small bib (0.99 cm²), an adult with a medium bib (1.50 cm²), an adult with a large bib (2.02 cm²). The scale bar in red (0.5 cm) indicates the scale of each photo, also given by the ruler on the left.

**
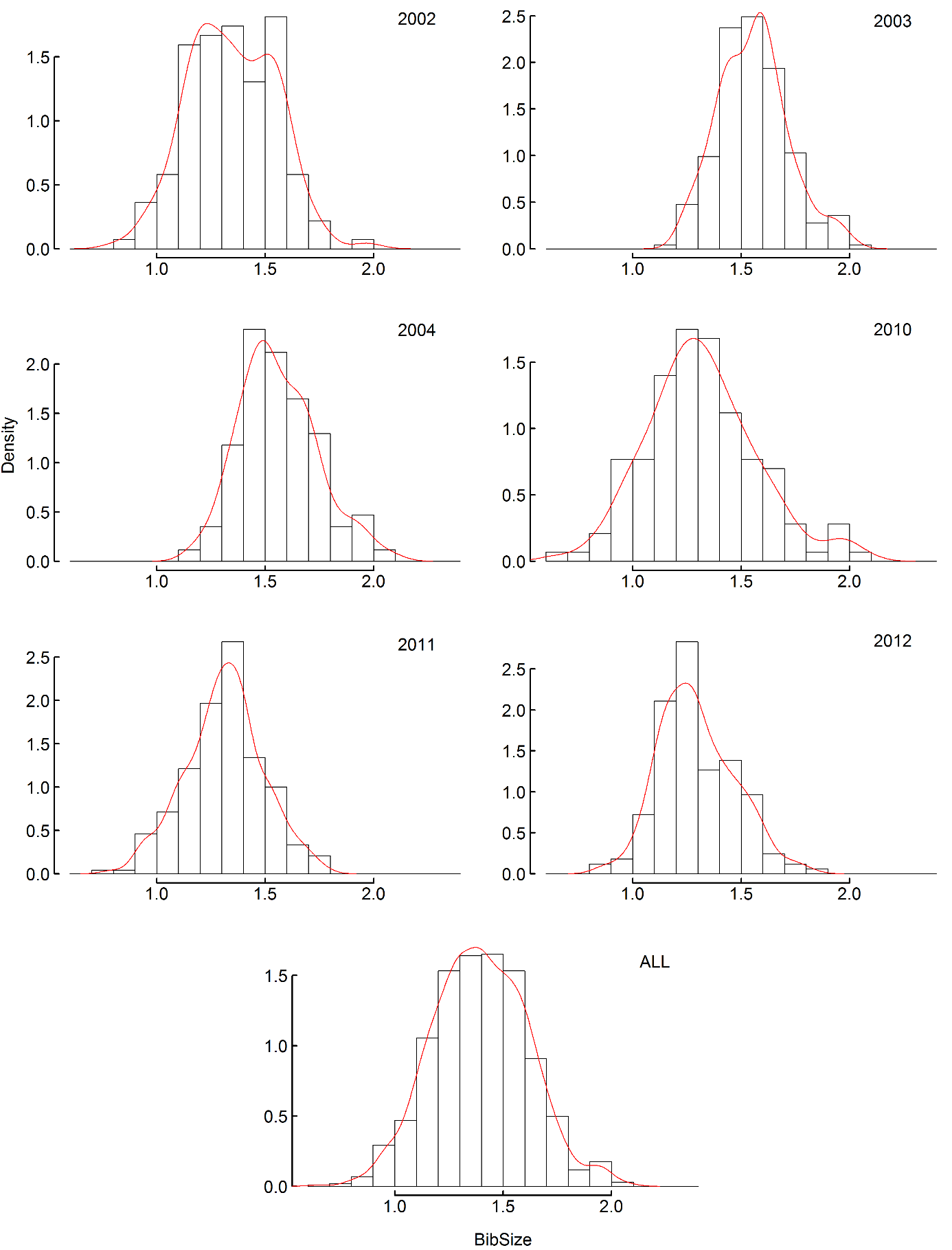
**

**Figure S2.** Distribution of bib size within each of the six years in which the photographs were taken, and pooled across all years.

**Appendix S2:** **Details on the within-individual centering approach**

In this paper, we used the within-individual centering approach (van de Pol & Wright 2009) to disentangle the between- and within-individual fixed effects of continuous predictor variables. This method consists in changing the standard mixed model equation used for model selection and cross-sectional analysis [a]:

**[a]**  $y_{ij}=\mu_{0}+\beta_{1}x_{ij}+u_{0j}+e_{0ij}$

where $y_{ij}$ is the study trait value for individual *j* at the *i*^th^ it was seen; $x_{ij}$ is the predictor variable value for individual *j* at the *i*^th^ hit was seen; $\mu_{0}$ is the overall intercept; $\beta_{1}$ is the combined within- and between-subject effect of *x* on *y,* $u_{0j}$ is the random effect of individual identity on the overall intercept and $e_{0ij}$ is the random noise of the observation.

It involves (i) the deviation from the individual mean value of the predictor variable for each observation $x_{ij}-\bar{x}_{j}$ to describe the within-individual variation component, and (ii) the individual mean value $\bar{x}_{j}$ to express the between-individual variation component [b]:

**[b]**  $y_{ij}=\mu_{0}+\beta_{w}(x_{ij}-\bar{x}_{j})+\beta_{b}\bar{x}_{j}+u_{0j}+e_{0ij}$

where $\beta_{w}$ is the within-subject effect of *x* on *y*; $\beta_{b}$ is the between-subject effect of *x* on *y*.

These two new predictor variables allow separately estimating and testing the significance of the within- and between-individual effects. A rewriting of this latter equation allows to test whether these two effects are statistically different from each other [c]:

**[c]**  $y_{ij}=\mu_{0}+\beta_{w}x_{ij}+{(\beta_{b}- \beta}_{w})\bar{x}_{j}+u_{0j}+e_{0ij}$

Obviously, other fixed or random effects can be added and were added in these analyses.

Extensive information about the within-subject centering approach can be found in van de Pol & Wright (2009).

**Appendix S3:** **Details on the selection of models describing bib size variability**

Detailed p-values for non-significant effects, before they were dropped during model selection, are as follows: P_MCMC_ = 0.3921 for age², P_MCMC_ = 0.3296 for sex × mass, and P_MCMC_ = 0.2770 for sex × age, P_MCMC_ = 0.6083 for sex × colony size (following the order of exclusion, sex × colony size was the first term removed and age² the last one).

The following table gives the AICc values, AICc weights and R² of the different models implied in model selection for the study of the variability in bib size (Table S3).

**Table S3.** Details on model selection for the study of bib size variability with AICc and R² values. R²_M_ is the proportion of variance explained by fixed effects and R²_C_ is the proportion of variance explained by the fixed and the random effects together. The minimum adequate model appears in bold. The final best model is in bold and underlined. We used “col.” as an abbreviation for “colony”, and “ind.” for “individual”.

| Fixed effects | Random effects | AICc | ΔAICc | AICcWt | R²_M_ | R²_C_ |
| --- | --- | --- | --- | --- | --- | --- |
| sex, mass, tarsus, col. size, age, age², age×sex, mass×sex, col. size×sex | sex\|year, col., ind. | -464.52 | 9.79 | 0.01 | 0.065 | 0.491 |
| sex, mass, tarsus, col. size, age, age², age×sex, mass×sex, col. size×sex | year, col., ind. | -463.60 | 10.71 | 0.00 | 0.059 | 0.530 |
| sex, mass, tarsus, col. size, age, age², age×sex, mass×sex, col. size×sex | col., ind. | -295.32 | 178.99 | 0.00 | 0.052 | 0.413 |
| sex, mass, tarsus, col. size, age, age², age×sex, mass×sex, col. size×sex | year, ind. | -448.78 | 25.53 | 0.00 | 0.058 | 0.514 |
| sex, mass, tarsus, col. size, age, age², age×sex, mass×sex | year, col., ind. | -465.53 | 8.78 | 0.01 | 0.059 | 0.531 |
| sex, mass, tarsus, col. size, age, age², mass×sex | year, col., ind. | -466.36 | 7.95 | 0.01 | 0.058 | 0.529 |
| sex, mass, tarsus, col. size, age, age² | year, col., ind. | -467.61 | 6.70 | 0.02 | 0.058 | 0.529 |
| **sex, mass, tarsus, col. size, age** | **year, col., ind.** | **-468.76** | **5.55** | **0.04** | **0.057** | **0.526** |
| sex, mass, tarsus, age | year, col., ind. | -467.17 | 7.13 | 0.02 | 0.055 | 0.517 |
| mass, tarsus, age, col. size | year, col., ind. | -464.65 | 9.66 | 0.01 | 0.053 | 0.522 |
| sex, mass, tarsus, col. size | year, col., ind. | -454.77 | 19.54 | 0.00 | 0.042 | 0.535 |
| sex, col. size, age | year, col., ind. | -440.18 | 34.13 | 0.00 | 0.036 | 0.495 |
| sex, mass, tarsus, col. size, log(age) | year, col., ind. | -471.41 | 2.90 | 0.16 | 0.060 | 0.535 |
| **sex, mass, tarsus, col. size, age ≤ 17 month, age > 17 month.** | **year, col., ind.** | **-474.31** | **0.00** | **0.67** | **0.063** | **0.542** |
| sex, mass, tarsus, col. size, age ≤ 17 month, age > 17 month. | year, col., ind. | -468.97 | 5.34 | 0.05 | 0.058 | 0.536 |

**Appendix S4:** **Details on the models describing survival according to bib size**

The following tables present all the CR models tested to study the relation between bib size and survival. Table S4 gives the results obtained with the main set of models presented in the parts Materials & Methods and Results of this paper, which contains all the individuals included in this study. Table S5 gives the results obtained with the additional set of models mentioned in part Discussion, which contains only individuals older than one year old. These latter models were developed to test an alternative hypothesis presented in part Discussion. They gave similar trends as what we obtained with the first set of models presented in Table S4 (a positive quadratic relation between survival and bib size) but with a limited significance that can be attributed to the decrease of the size of the data set (from 662 to 487 individuals with much less repetitions: 582 observations) and also there might be a smaller link between survival and bib size for older individuals. The main prediction of the alternative hypothesis is a positive linear relationship between survival probability and bib size among individuals younger than one year old. This prediction was not verified. According to these findings we could reject this alternative hypothesis. Furthermore, in this additional set of models, there was no effect of time on survival. Thus no interaction between time and bib size or time and sex was tested.

To further inform the discussion, it may be interesting to note the following. The significant association between survival and SB indicates that viability selection is acting on the value of the trait perceived by the individuals at each time step. At each time step, the trait expressed by the individual (SB) may deviate from MAB due to the environment, but these changes are variations around the expected value. In agreement, MAB and SB are strongly correlated (Pearson correlation coefficient: *r* = 0.84, 95%CI = [0.82,0.86]) indicating that trait expression is not variable enough within individuals to substantially change their bib size expression in the population over their capture history (i.e. small bibs tend to stay small, medium to stay medium and large to stay large). The fact that we obtained the same pattern of disruptive selection for SB and MAB supports the idea that an individual globally experiences the same selective pressure over life.

**Table S4.** Summary of the main set of capture-recapture models. All the individuals were included. MAB is the mean adjusted bib size and SB is the standardized bib size, SR is the raw bib size (i.e. the untransformed measure obtained from photos taken in the field). ΔAICc is used to compare any model with the final best model while Δ_0_AICc is used to compare any model with the ‘null model’ (i.e. the best model without any effect of bib size on survival, Φ_t+sex_,p_t_). The rank gives the descending order of AICc among the models presented in this table. In each subpart, the models are listed in ascending order of AICc. AICcW is the AICc weights, calculated among the models presented in this table. 1st(...) = effect present only during first year of capture-recapture history (i.e. after the first photograph was taken). The notation used is the general notation of Lebreton et al. (1992).

| Model | AICc | Deviance | *K* | ΔAICc | Δ_0_AICc | Rank | AICcW |
| --- | --- | --- | --- | --- | --- | --- | --- |
| Φ_t·MAB²+sex,_p_t_ | 1871,1 | 1822,1 | 24 | 0,0 | -10,1 | 1 | 0,66 |
| Φ_t·MAB²+sex·MAB²,_p_t_ | 1874,5 | 1821,3 | 26 | 3,4 | -6,7 | 2 | 0,12 |
| Φ_t+sex+MAB²,_p_t_ | 1875,6 | 1841,0 | 17 | 4,5 | -5,6 | 3 | 0,07 |
| Φ_t+sex·MAB²,_p_t_ | 1876,9 | 1840,3 | 18 | 5,8 | -4,3 | 4 | 0,04 |
| Φ_t+sex+MAB+MAB²,_p_t_ | 1877,2 | 1840,7 | 18 | 6,1 | -4,0 | 5 | 0,03 |
| Φ_t+sex+1st(SB²),_p_t_ | 1878,4 | 1843,9 | 17 | 7,3 | -2,8 | 6 | 0,02 |
| Φ_t+sex+1st(RB²),_p_t_ | 1880,1 | 1845,6 | 17 | 9,0 | -1,1 | 7 | 0,01 |
| Φ_t+sex·1st(SB²),_p_t_ | 1880,2 | 1843,6 | 18 | 9,1 | -1,0 | 8 | 0,01 |
| Φ_t+sex+1st(SB+SB²),_p_t_ | 1880,2 | 1843,6 | 18 | 9,1 | -1,0 | 9 | 0,01 |
| Φ_t+sex·(MAB+MAB²),_p_t_ | 1880,2 | 1839,5 | 20 | 9,1 | -1,0 | 10 | 0,01 |
| Φ_t+sex·1st(RB²),_p_t_ | 1880,8 | 1844,2 | 18 | 9,7 | -0,4 | 11 | 0,01 |
| Φ_t+sex,_p_t_  (‘*null model’)* | 1881,2 | 1848,7 | 16 | 10,1 | 0,0 | 12 | 0,00 |
| Φ_t+sex+1st(RB+RB²),_p_t_ | 1882,0 | 1845,4 | 18 | 10,9 | 0,8 | 13 | 0,00 |
| Φ_t+sex+SB,_p_t_ | 1882,3 | 1847,8 | 17 | 11,2 | 1,1 | 14 | 0,00 |
| Φ_t+sex+SB²,_p_t_ | 1882,4 | 1847,9 | 17 | 11,3 | 1,2 | 15 | 0,00 |
| Φ_t+sex+SB+SB²,_p_t_ | 1882,6 | 1846,0 | 18 | 11,5 | 1,4 | 16 | 0,00 |
| Φ_t·1st(SB)+sex,_p_t_ | 1882,6 | 1839,8 | 21 | 11,5 | 1,4 | 17 | 0,00 |
| Φ_t+sex+RB,_p_t_ | 1882,8 | 1848,3 | 17 | 11,7 | 1,6 | 18 | 0,00 |
| Φ_t+sex,_p_t+sex_ | 1883,2 | 1848,7 | 17 | 12,1 | 2,0 | 19 | 0,00 |
| Φ_t+sex+RB²,_p_t_ | 1883,2 | 1848,7 | 17 | 12,1 | 2,0 | 20 | 0,00 |
| Φ_t+sex+MAB,_p_t_ | 1883,2 | 1848,7 | 17 | 12,1 | 2,0 | 21 | 0,00 |
| Φ_t+sex+1st(RB),_p_t_ | 1883,2 | 1848,7 | 17 | 12,1 | 2,0 | 22 | 0,00 |
| Φ_t+sex+1st(SB),_p_t_ | 1883,2 | 1848,7 | 17 | 12,1 | 2,0 | 23 | 0,00 |
| Φ_t·(MAB+MAB²)+sex,_p_t_ | 1883,8 | 1820,1 | 31 | 12,7 | 2,6 | 24 | 0,00 |
| Φ_t+sex·1st(SB+SB²),_p_t_ | 1884,1 | 1843,4 | 20 | 13,0 | 2,9 | 25 | 0,00 |
| Φ_t+sex·MAB,_p_t_ | 1884,3 | 1847,7 | 18 | 13,2 | 3,1 | 26 | 0,00 |
| Φ_t+sex+RB+RB²,_p_t_ | 1884,5 | 1847,9 | 18 | 13,4 | 3,3 | 27 | 0,00 |
| Φ_t+sex·1st(RB),_p_t_ | 1884,8 | 1848,2 | 18 | 13,7 | 3,6 | 28 | 0,00 |
| Φ_t+sex·1st(RB+RB²),_p_t_ | 1884,9 | 1844,2 | 20 | 13,8 | 3,7 | 29 | 0,00 |
| Φ_t·1st(SB+SB²)+sex,_p_t_ | 1884,9 | 1833,8 | 25 | 13,8 | 3,7 | 30 | 0,00 |
| Φ_t+sex·1st(SB),_p_t_ | 1885,0 | 1848,5 | 18 | 13,9 | 3,8 | 31 | 0,00 |
| Φ_t·1st(SB²)+sex·1st(SB²),_p_t_ | 1886,8 | 1839,8 | 23 | 15,7 | 5,6 | 32 | 0,00 |
| Φ_t·1st(RB²)+sex,_p_t_ | 1886,8 | 1844,0 | 21 | 15,7 | 5,6 | 33 | 0,00 |
| Φ_t,_p_t_ | 1887,9 | 1857,5 | 15 | 16,8 | 6,7 | 34 | 0,00 |
| Φ_t·(MAB+MAB²)+sex·(MAB+MAB²),_p_t_ | 1888,3 | 1816,1 | 35 | 17,2 | 7,1 | 35 | 0,00 |
| Φ_t·1st(RB)+sex,_p_t_ | 1889,2 | 1846,4 | 21 | 18,1 | 8,0 | 36 | 0,00 |
| Φ_t·1st(SB)+sex,_p_t_ | 1889,5 | 1846,7 | 21 | 18,4 | 8,3 | 37 | 0,00 |
| Φ_t,_p_t+sex_ | 1889,6 | 1857,1 | 16 | 18,5 | 8,4 | 38 | 0,00 |
| Φ_t·1st(RB²)+sex·1st(RB²),_p_t_ | 1890,4 | 1843,4 | 23 | 19,3 | 9,2 | 39 | 0,00 |
| Φ_t,_p_._ | 1891,1 | 1873,0 | 9 | 20,0 | 9,9 | 40 | 0,00 |
| Φ_t·sex·1st(SB),_p_t_ | 1892,5 | 1822,4 | 34 | 21,4 | 11,3 | 41 | 0,00 |
| Φ_t·MAB+sex,_p_t_ | 1892,5 | 1843,5 | 24 | 21,4 | 11,3 | 42 | 0,00 |
| Φ_t·1st(RB)+sex·1st(RB),_p_t_ | 1893,1 | 1846,1 | 23 | 22,0 | 11,9 | 43 | 0,00 |
| Φ_t·sex·1st(SB+SB²),_p_t_ | 1893,1 | 1795,2 | 47 | 22,0 | 11,9 | 44 | 0,00 |
| Φ_t·1st(SB)+sex·1st(SB),_p_t_ | 1893,4 | 1846,5 | 23 | 22,3 | 12,2 | 45 | 0,00 |
| Φ_t·sex·MAB²,_p_t_ | 1893,7 | 1810,9 | 40 | 22,6 | 12,5 | 46 | 0,00 |
| Φ_t·1st(RB+RB²)+sex,_p_t_ | 1893,9 | 1840,7 | 26 | 22,8 | 12,7 | 47 | 0,00 |
| Φ_t·1st(SB+SB²)+sex·1st(SB+SB²),_p_t_ | 1895,2 | 1833,6 | 30 | 24,1 | 14,0 | 48 | 0,00 |
| Φ_t·MAB+sex·MAB,_p_t_ | 1895,8 | 1842,6 | 26 | 24,7 | 14,6 | 49 | 0,00 |
| Φ_t·sex·1st(RB²),_p_t_ | 1896,5 | 1826,5 | 34 | 25,4 | 15,3 | 50 | 0,00 |
| Φ_.,_p_t_ | 1897,5 | 1879,4 | 9 | 26,4 | 16,3 | 51 | 0,00 |
| Φ_t·sex·1st(SB²),_p_t_ | 1898,8 | 1828,8 | 34 | 27,7 | 17,6 | 52 | 0,00 |
| Φ_t·sex·1st(RB),_p_t_ | 1899,3 | 1829,3 | 34 | 28,2 | 18,1 | 53 | 0,00 |
| Φ_t·1st(RB+RB²)+sex·1st(RB+RB²),_p_t_ | 1900,4 | 1838,9 | 30 | 29,3 | 19,2 | 54 | 0,00 |
| Φ_t·sex·1st(RB+RB²),_p_t_ | 1902,0 | 1804,1 | 47 | 30,9 | 20,8 | 55 | 0,00 |
| Φ_.,_p_._ | 1903,1 | 1899,1 | 2 | 32,0 | 21,9 | 56 | 0,00 |
| Φ_t·sex·(MAB+MAB²),_p_t_ | 1904,3 | 1786,8 | 56 | 33,2 | 23,1 | 57 | 0,00 |
| Φ_t·sex·MAB,_p_t_ | 1910,9 | 1828,1 | 40 | 39,8 | 29,7 | 58 | 0,00 |

**Table S5.** Summary of the additional set of capture-recapture models. Individuals younger than one year old were removed. MAB is the mean adjusted bib size and SB is the standardized bib size, SR is the raw bib size (i.e. the untransformed measure obtained from photos taken in the field). ΔAICc is used to compare any model with the final best model while Δ_0_AICc is used to compare any model with the ‘null model’ (i.e. the best model without any effect of bib size on survival, Φ_t+sex_,p_t_). The rank gives the descending order of AICc among the models presented in this table. In each subpart, the models are listed in ascending order of AICc. AICcW is the AICc weights, calculated among the models presented in this table. 1st(...) = effect present only during first year of capture-recapture history (i.e. after the first photograph was taken). The notation used is the general notation of Lebreton et al. (1992).

| Model | AICc | Deviance | *K* | ΔAICc | Δ_0_AICc | Δ_0_AICc | AICcW |
| --- | --- | --- | --- | --- | --- | --- | --- |
| Φ_sex+MAB²_,p_t_ | 1074.18 | 1051.83 | 11 | 0.00 | -0.87 | 1 | 0.21 |
| Φ_sex_,p_t_ | 1075.05 | 1054.76 | 10 | 0.87 | 0.00 | 2 | 0.14 |
| Φ_sex+MAB+MAB²_,p_t_ | 1075.42 | 1051.01 | 12 | 1.24 | 0.37 | 3 | 0.11 |
| Φ_sex+1st(RB²)_,p_t_ | 1075.61 | 1053.26 | 11 | 1.43 | 0.56 | 4 | 0.10 |
| Φ_sex+1st(SB²)_,pt | 1075.80 | 1053.45 | 11 | 1.62 | 0.74 | 5 | 0.09 |
| Φ_sex+MAB_,p_t_ | 1076.58 | 1054.23 | 11 | 2.40 | 1.53 | 6 | 0.06 |
| Φ_._,p_t_ | 1076.79 | 1058.55 | 9 | 2.61 | 1.74 | 7 | 0.06 |
| Φ_sex·MAB²_,p_t_ | 1077.14 | 1052.73 | 12 | 2.96 | 2.08 | 8 | 0.05 |
| Φ_t_,p_._ | 1077.24 | 1059.01 | 9 | 3.06 | 2.19 | 9 | 0.05 |
| Φ_sex+1st(SB)_,p_t_ | 1078.55 | 1056.20 | 11 | 4.37 | 3.50 | 10 | 0.02 |
| Φ _sex+1st(RB)_,p_t_ | 1078.66 | 1056.31 | 11 | 4.48 | 3.60 | 11 | 0.02 |
| Φ_sex·1st(SB²)_,p_t_ | 1079.94 | 1055.53 | 12 | 5.76 | 4.89 | 12 | 0.01 |
| Φ_sex·MAB_,p_t_ | 1079.95 | 1055.53 | 12 | 5.77 | 4.89 | 13 | 0.01 |
| Φ_sex·1st(RB²)_,p_t_ | 1080.01 | 1055.60 | 12 | 5.83 | 4.96 | 14 | 0.01 |
| Φ_sex·(MAB+MAB²)_,p_t_ | 1080.52 | 1051.97 | 14 | 6.34 | 5.47 | 15 | 0.01 |
| Φ_sex+1st(SB+SB²)_,p_t_ | 1080.49 | 1056.08 | 12 | 6.31 | 5.44 | 16 | 0.01 |
| Φ_sex·1st(RB²)_,p_t_ | 1080.52 | 1056.11 | 12 | 6.34 | 5.47 | 17 | 0.01 |
| Φ_sex+1st(RB+RB²)_,p_t_ | 1080.58 | 1056.17 | 12 | 6.40 | 5.53 | 18 | 0.01 |
| Φ_sex·1st(SB)_,p_t_ | 1080.61 | 1056.20 | 12 | 6.43 | 5.56 | 19 | 0.01 |
| Φ_t_p_t_ | 1081.92 | 1051.29 | 15 | 7.74 | 6.87 | 20 | 0.00 |
| Φ_t_p_t_ | 1081.92 | 1051.29 | 15 | 7.74 | 6.87 | 21 | 0.00 |
| Φ_sex·1st(RB+RB²)_,p_t_ | 1084.06 | 1055.50 | 14 | 9.88 | 9.00 | 22 | 0.00 |
| Φ_sex·1st(SB+SB²)_,p_t_ | 1084.06 | 1055.50 | 14 | 9.88 | 9.01 | 23 | 0.00 |
| Φ_._p_._ | 1086.40 | 1082.38 | 2 | 12.22 | 11.34 | 24 | 0.00 |

**
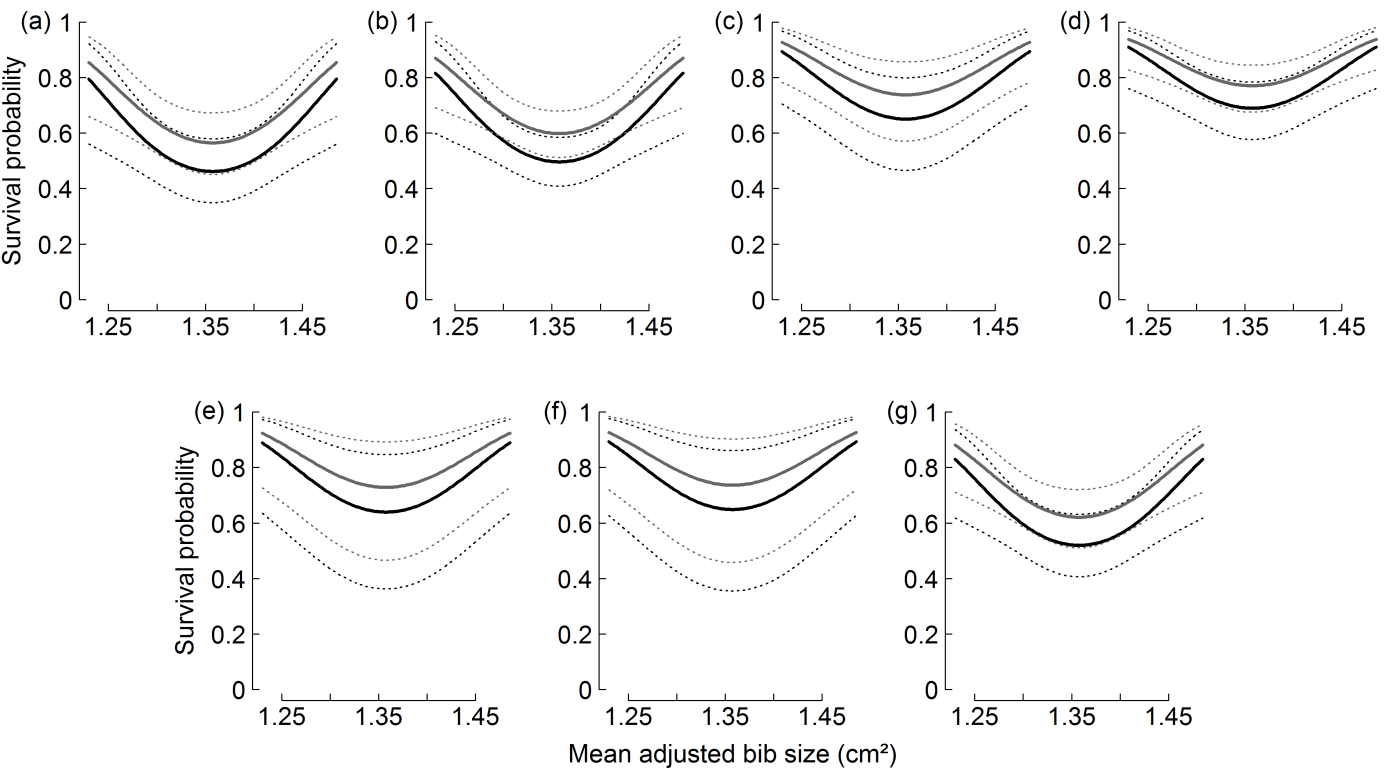
**

**Figure S3**. Survival probability according to mean adjusted bib size in each time step of the study: (a) 2002–2003, (b) 2003–2004, (c) 2004–2005, (d) 2005–2008, (e) 2008–2009, (f) 2009–2010, (g) 2010–2011. Survival is confounded with recapture probability for the last time step (2011–2012) and thus unidentifiable. The plotted lines represent estimated survival probabilities obtained with the best model *without* interaction between time and bib size (ϕ_t+MAB²+sex_,ρ_t_) which indicates a significant pattern of disruptive viability selection over the dataset. Females are plotted in black and males in grey. The solid lines indicate the means and dotted lines 95% confidence intervals.

**
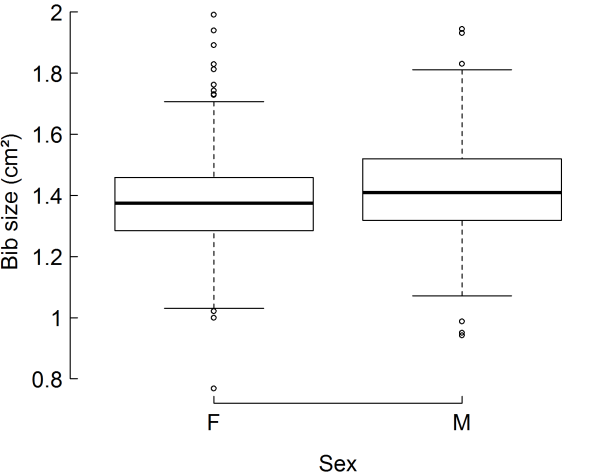
**
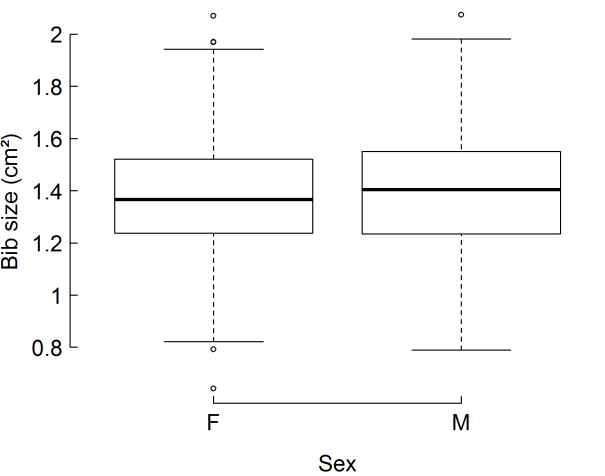


**Figure S4.** Distribution of bib size in females (F) and males (M). On the left, bib size *was adjusted* by the other effects of the predictor variables included in the linear mixed model (see main text). On the right, bib size *was not adjusted* by any other effects.

**References**

Lebreton, J.-D., Burnham, K.P., Clobert, J. & Anderson, D.R. (1992) Modeling Survival and Testing Biological Hypotheses Using Marked Animals: A Unified Approach with Case Studies. *Ecological Monographs*, **62**, 67–118.

van de Pol, M. & Wright, J. (2009) A simple method for distinguishing within- versus between-subject effects using mixed models. *Animal Behaviour*, **77**, 753–758.
